# Laminar architecture of visual and auditory responses in the supplementary eye field of macaques

**DOI:** 10.1093/cercor/bhag064

**Published:** 2026-06-19

**Authors:** Pranavan Thirunavukkarasu, Steven P Errington, Amirsaman Sajad, Benjamin W Corrigan, Jeffrey D Schall

**Affiliations:** Centre for Integrative and Applied Neuroscience, Centre for Vision Research, Department of Biology, York University, 4700 Keele Street, Toronto, ON M3J 1P3, Canada; Biosciences Institute, Faculty of Medical Sciences, Newcastle University, Tyne and Wear, Newcastle upon Tyne NE1 7RU, United Kingdom; International Center for Primate Brain Research, Center for Excellence in Brain Science and Intelligence Technology, Chinese Academy of Sciences, No. 500 Qiang Ye Road, Songjiang District, Shanghai 201602, P.R. China; Centre for Integrative and Applied Neuroscience, Centre for Vision Research, Department of Biology, York University, 4700 Keele Street, Toronto, ON M3J 1P3, Canada; Centre for Integrative and Applied Neuroscience, Centre for Vision Research, Department of Biology, York University, 4700 Keele Street, Toronto, ON M3J 1P3, Canada

**Keywords:** auditory response, current-source density, laminar architecture, supplementary eye field, visual response

## Abstract

We describe properties and laminar organization of visual and auditory responses sampled with linear electrode arrays in and around the supplementary eye field of four macaque monkeys performing a visually guided task with a tone secondary reinforcer. Of pyramidal neurons in all layers and interneurons in L3 and L5/6, one-quarter were visually responsive, one-quarter, auditory, with 30% bimodal. Visual neurons were less common rostral, lateral, and medial to the supplementary eye field. Facilitated visual and auditory responses were much more common than suppressed. Pyramidal and interneurons responded to the stimulus in either hemifield with a weak bias for contralateral targets. Visual response latencies were comparable to values previously reported and later than auditory responses. Facilitated visual responses of interneurons preceded those of pyramidal neurons. Transient visual responses preceded sustained. Current-source density revealed visually evoked current sinks after auditory sinks that were transient for bright flashes and prolonged for the discrete task target. The earliest sink appeared at the L3 to L5 border and spread superficially and deeper with idiosyncratic variability across monkeys. These findings reveal new details about sensory processing in medial frontal cortex and complement our previous descriptions of the functional properties and laminar organization of neurons supporting cognitive control.

## Introduction

This report continues a series of investigations of the laminar organization of functional types of neurons in SEF, an agranular area on the dorsomedial convexity in macaques. Previously, we described the laminar organization of neurons’ signaling error, reward gain and loss, as well as conflict, event timing, and goal maintenance during a saccade-countermanding task (Sajad et al. 2019; Sajad et al. 2022). Here, we describe the laminar organization and properties of visually and acoustically responsive neurons sampled during performance of a saccade-countermanding task. Many publications have described visual (Schlag and Schlag-Rey 1987; Schall 1991a, 1991b; Russo and Bruce 1993; Russo and Bruce 1996; Schlag-Rey et al. 1997; Russo and Bruce 2000; Roesch and Olson 2003; Nakamura et al. 2005; Pouget et al. 2005; Shichinohe et al. 2009; Purcell et al. 2012; Bharmauria et al. 2021; Schütz et al. 2023) and auditory (Schall 1991a) responses in the supplementary eye field (SEF), but other than our earlier publication describing passive visual responses (Godlove et al. 2014), nothing is known about the laminar organization of task-related visual or auditory responses. The new observations provide further evidence that the laminar organization of agranular cortex differs from that of sensory areas (Godlove et al. 2014; Ninomiya et al. 2015) and complement earlier descriptions of the laminar organization of neurons’ signaling errors, reward gain and loss, conflict, timing of events, and goal maintenance (Sajad et al. 2019, 2022). Knowing how SEF processes visual and auditory information is necessary to understand how environmental events are integrated into cognitive control.

## Methods

All data acquisition procedures were approved by the Vanderbilt Institutional Animal Care and Use Committee (Protocol M1700067) in accordance with the United States Department of Agriculture and Public Health Service Policy on Humane Care and Use of Laboratory Animals.

### Data acquisition: stop-signal task

**Figure 1:**
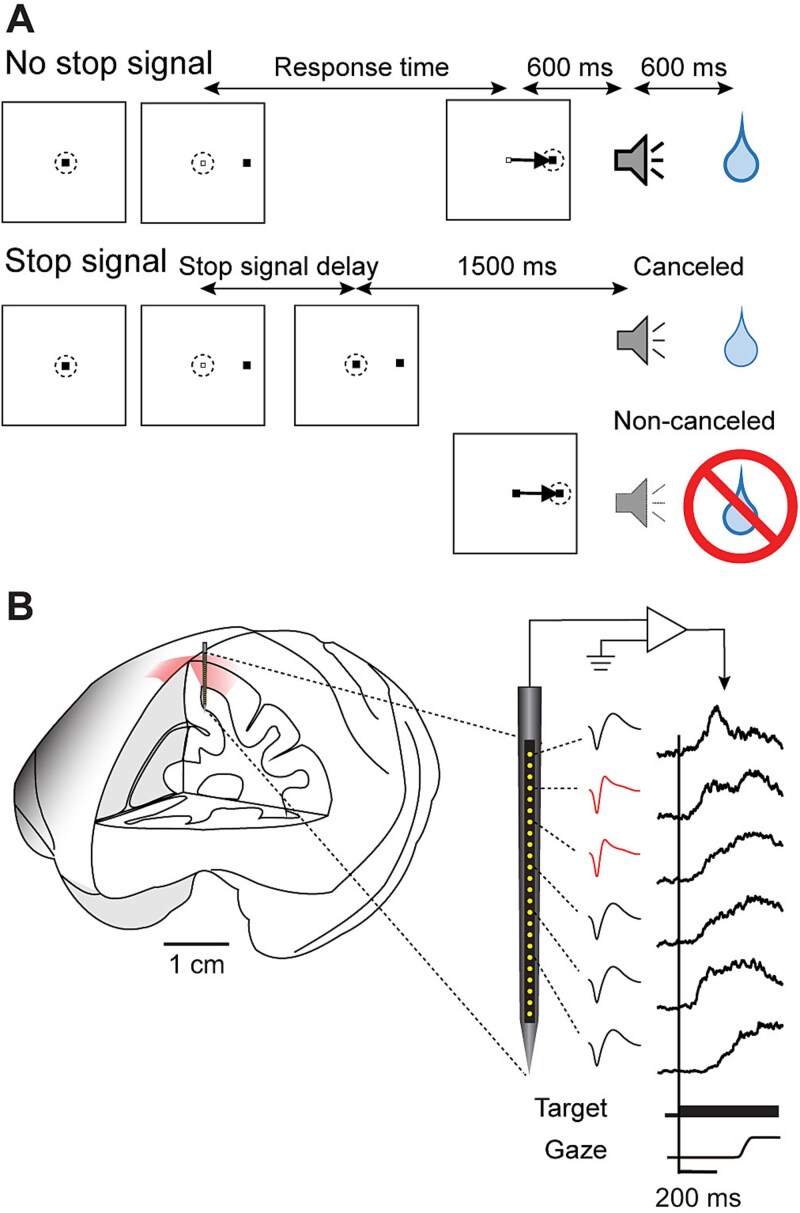
Experimental design. (A) Visually guided saccade-countermanding task. Monkeys initiated trials by fixating on a central point. After a variable time, the center of the fixation point was extinguished. A visual target was presented simultaneously at one of two possible locations. On no-stop-signal trials, monkeys were required to shift gaze to the target, whereupon after 600 (X, Eu) or 700 (Da, Jo) ± 0 ms, a high-pitched auditory feedback tone was delivered, and 530 (Da, Jo) or 600 (X, Eu) ± 0 ms later fluid reward was provided. On stop-signal trials (~40% of trials), after the target appeared, the center of the fixation point was re-illuminated after a variable stop-signal delay, which instructed the monkey to cancel the saccade, in which case the same high-pitched tone was presented after a 1,500 ± 0 ms hold time followed, after 530 or 600 ± 0 ms, by fluid reward. Stop-signal delay was adjusted such that monkeys successfully canceled the saccade in ~ 50% of trials. In the remaining trials, monkeys made noncancelled errors, which were followed after 600 or 700 ± 0 ms by a low-pitched tone, and no reward was delivered. Monkeys could not initiate trials earlier after errors. (B) Neural spiking was recorded across all layers of SEF using a linear electrode array. Neurons with both broad (black) and narrow (red) spikes were sampled. Spiking modulation shown here was measured relative to presentation of the visual target.

The saccade stop-signal (countermanding) task utilized in this study has been used previously (Hanes and Schall 1995; Hanes and Carpenter 1999; Cabel et al. 2000; Colonius et al. 2001; Kornylo et al. 2003; Morein-Zamir and Kingstone 2006; Walton and Gandhi 2006; Thakkar et al. 2011; Thakkar et al. 2015; Godlove and Schall 2016; Wattiez et al. 2016; Verbruggen et al. 2019). Briefly, trials were initiated when monkeys fixated at a central point (Fig. 1A). Following a variable time period, the center of the fixation point was removed leaving an outline. At this point, a peripheral target was presented simultaneously on either the left or right hand of the screen.

On trials with no-stop signal, monkeys were rewarded for shifting gaze to the target (no-stop trials). On a proportion of random trials (35% to 45%), a stop signal was presented through re-illumination of the center of the fixation point, and monkeys were rewarded for withholding the saccade and maintaining fixation on the fixation spot. The stop signal appeared after a variable stop-signal delay (SSD) after the target. An initial set of SSDs, separated by either 40 or 60 ms, were selected for each recording session to yield ~ 50% correct inhibition. The delay was then manipulated through an adaptive staircasing procedure in which stopping difficulty was based on performance. When monkeys failed to inhibit a response, the SSD was decreased by a random step to increase the likelihood of success on the next stop trial. Similarly, when monkeys were successful in their inhibition, the SSD was increased to reduce the likelihood of success on the next stop trial. This procedure was employed to ensure that subjects failed to inhibit action on ~50% of all stop-signal trials. Following a correct response, an auditory tone was sounded after 600 (X, Eu) or 700 (Da, Jo) ± 0 ms interval, followed after another 600 (X, Eu) or 530 (Da, Jo) ± 0 ms interval by fluid reward. All monkeys exhibited typical countermanding behavior, details of which have been previously reported for Eu and X (Sajad et al. 2019, 2022) and Da and Jo (Corrigan et al. 2024).

### Data acquisition: animal care, surgical, and neurophysiology procedures

Data were collected from two male bonnet macaques (Eu, *Macaca radiata*, 8.8 kg; Da, *M. radiata*, 9.5 kg), one male rhesus macaque (Jo, *Macaca mulatta,* 13.8 kg), and one female rhesus macaque (X, *M. mulatta*, 6.0 kg) performing a saccade-countermanding task (Hanes and Schall 1995; Sajad et al. 2019, 2022). All procedures were approved by the Vanderbilt Institutional Animal Care and Use Committee in accordance with the United States Department of Agriculture and Public Health Service Policy on Humane Care and Use of Laboratory Animals.

Surgical details have been described previously (Godlove et al. 2011). Magnetic resonance images (MRIs) were obtained with a Philips Intera Achieva 3-T scanner using SENSE Flex-S surface coils positioned above or below the head. High-resolution T1-weighted structural images were acquired with a 3D turbo field echo sequence (Repetition time (TR) = 8.729 ms; 130 slices; 0.70 mm thickness). These images were used to guide placement of Cilux recording chambers (Crist Instruments) over the medial frontal cortex. Chambers were implanted normal to the cortical surface and centered relative to stereotaxic landmarks. For monkey Eu, the chamber was angled 17° relative to stereotaxic vertical, centered on the midline, and positioned 30 mm anterior to the interaural line. For monkey X, the chamber was angled 9° relative to stereotaxic vertical, centered on the midline, and positioned 28 mm anterior to the interaural line. For monkey Da, the chamber was implanted normal to the cortex, centered on the midline, and positioned 30 mm anterior to the interaural line. For monkey Jo, the chamber was implanted 28° relative to stereotaxic horizontal and 19° relative to stereotaxic vertical, positioned 1.2 mm lateral to the midline, and 33.3 mm anterior to the interaural line.

Chambers implanted over the medial frontal cortex were mapped using tungsten microelectrodes (2 to 4 MΩ; FHC, Bowdoin, ME) to apply 200-ms trains of biphasic micro-stimulation (333 Hz, 200 μs pulse width). The general location of SEF was identified as the area from which saccades could be elicited using < 50 μA of current (Schall 1991b; Martinez-Trujillo et al. 2004). In monkeys Eu, Jo, and X, the SEF chamber was placed over the left hemisphere, whereas in monkey Da the SEF chamber was placed over both hemispheres.

Neural spiking across the cortical layers was sampled at 44 different sites in the dorsomedial frontal cortex (Da: 21, Eu:2, Jo: 18, X: 3). The laminar depth profiles of three of these penetration sites (Eu 1; X 2) were previously reported and confirmed to be perpendicular to the cortex verified through co-registered CT/MRI matching (Godlove et al. 2014; Sajad et al. 2019, 2022). In monkey Eu, the perpendicular penetrations sampled activity at sites located 5 mm lateral to the midline and 31 mm anterior to the interaural line. In monkey X, the perpendicular penetrations sampled activity located 5 mm lateral to the midline and 29 and 30 mm anterior to the interaural line, respectively. Here, we report results from more sites in monkeys Eu and X plus many more samples from the other two monkeys. The other sites in Eu and X were adjacent to those reported previously, so the curvature of the dorsomedial convexity was not much different. The sites reported from the other two monkeys were also on the dorsomedial convexity. In monkey Da, the perpendicular penetrations sampled activity at sites located from −5 to −2 mm and 3 to 5 mm lateral to the midline (spanning left and right hemispheres respectively) and from 27 to 33 mm anterior to the interaural line. In monkey Jo, the perpendicular penetrations sampled activity at sites located from −4 to −1 mm lateral to the midline (spanning left hemisphere) and from 28 to 32 mm anterior to the interaural line. The physiological characteristics of the penetrations were consistent with perpendicular penetrations. Locations where penetrations were not perpendicular were identified by sampling medial and lateral to SEF.

Spiking activity and local field potentials were recorded from monkeys Eu and X using 24-channel Plexon U-probes (Dallas, TX) with a 150-μm interelectrode spacing allowing sampling from all layers, whereas for monkeys Da and Jo, 32-channel NeuroNexus probes (Ann Arbor, MI) with 50-μm, 100-μm, and 150-μm spacing were used (Fig. 1B). The U-probes were 100 mm in length with 30 mm reinforced tubing, 210 μm probe diameter, 30° tip angle, and had 500 μm between the tip and first contact. Contacts were referenced to the probe shaft and grounded to the headpost. For U-probes, we used custom-built guide tubes consisting of 26-gauge polyether ether ketone (PEEK) tubing (Plastics One, Roanoke, VA) cut to length and glued into 19-gauge stainless-steel hypodermic tubing (Small Parts Inc., Logansport, IN). For NeuroNexus arrays, probes were housed within custom-built guide tubes made from 21-gauge stainless-steel hypodermic tubing (Small Parts Inc., Logansport, IN). This tubing had been cut to length, deburred, and polished so that they effectively support the probes as they penetrated dura and entered cortex. The stainless-steel guide tube provided mechanical support, while the PEEK tubing electrically insulated the shaft of the probes, and provided an inert, low-friction interface that aided in loading and penetration. For both intracortical electrodes, contacts were referenced to the guide tube in which they were housed and grounded to the headpost.

Microdrive adapters were fit to recording chambers with < 400 μm of tolerance and locked in place at a single radial orientation (Crist Instruments, Hagerstown, MD). After setting up hydraulic microdrives (FHC, Bowdoin, ME; Narishige, Tokyo, Japan) on these adapters, pivot points were locked in place by means of a custom mechanical clamp. Neither guide tubes nor probes were removed from the microdrives once recording commenced within a single monkey. These methods ensured that we were able to sample neural activity from precisely the same location relative to the chamber on repeated sessions.

For monkeys Eu and X, electrophysiology data were processed with unity-gain high-input impedance head stages (HST/32o25-36P-TR; Plexon). All data were streamed to a single data acquisition system (MAP; Plexon). Time stamps of trial events were recorded at 500 Hz. Eye position data were streamed to the Plexon computer at 1 kHz using an EyeLink 1000 infrared eye-tracking system (SR Research, Kanata, ON, Canada). For monkeys Da and Jo, electrodes were connected to the recording system through high-input impedance ZIF-clip head stages (ZD32; Tucker-Davis Technologies, Alachua, FL), and signals were digitized through a preamplifier (PZ5; Tucker-Davis Technologies) and streamed to a single data acquisition system (TDT System 3; Tucker-Davis Technologies) at 24,414.0625 Hz. Neural data were filtered online through the software Synapse (Tucker-Davis Technologies). Local field potentials were bandpass filtered between 3 and 300 Hz, with notch filters at 60, 120, and 180 Hz. Spiking data were derived from a broadband signal, bandpass filtered between 300 and 5,000 Hz.

An identical daily recording protocol across monkeys and sessions was carried out. For monkeys Eu and X, the animal sat in an enclosed primate chair with their head restrained 45 cm from a CRT monitor (Dell P1130, background luminance of 0.10 cd/m^2^, refresh rate 70 Hz). The screen subtended 46° × 36° of the visual angle. For monkeys Da and Jo, the animal sat in an enclosed primate chair with their head restrained 57 cm from a CRT monitor (NEC MultiSync FE922-BK, background luminance of 0.10 cd/m^2^, refresh rate 60 Hz). Eye position data for all animals were collected at 1 kHz using an EyeLink 1000 infrared eye-tracking system (SR Research). For monkeys Eu and X, behavioral and neural was streamed to a MAP single data acquisition system (MAP), whereas for monkeys Da and Jo, this was streamed to a TDT System 3 single data acquisition system (TDT System 3; Tucker-Davis Technologies) and amalgamated with other behavioral and neurophysiological data.

After advancing the electrode arrays to the desired depth, they were allowed to settle until recordings stabilized across contacts. The settling time was 69 ± 25 min (range: 30 to 180 min) for monkeys Da and Jo, and 180 to 240 min for monkeys Eu and X, resulting in consistently stable recordings. For Eu and X, once these recordings stabilized, an hour of resting-state activity in near-total darkness was recorded. This was followed by the passive presentation of visual flashes followed by periods of total darkness in alternating blocks. Results from this passive viewing protocol have been previously reported (Godlove et al. 2014). Finally, the monkey then performed ~ 2,000 to 3,000 trials of the saccade-countermanding (stop-signal) task. Single units for all animals were sorted online using a software window discriminator and for Eu and X were refined offline using principal component analysis implemented in Plexon Offline Sorter. For monkeys Da and Jo, both single units and multiunit activity were extracted using automated spike sorting via Kilosort2.5.

### Data analysis: spike rate modulation and statistics

Two approaches were employed. The first detected sensory response in spike density functions calculated across trials. The second detected sensory responses in single trials based on deviations from Poisson statistics. They will be explained in turn.

Across trials, spike density functions were calculated by convolving spike trains with a kernel that approximates a postsynaptic potential such that SDF(*t*) = (1 − exp(−*t*/τ_g_))*exp(−*t*/τ_d_), where the growth time constant (τ_g_) was set to 1 ms and the decay constant (τ_d_), to 20 ms. Across trials, significant spike rate modulation for visual responses, facilitated or suppressed, was defined as spike densities that exceeded 3 SD from a baseline period (−250 to 0 ms relative to target) for longer than 50 ms and reached a magnitude of 6 SD for at least 25 ms. For auditory responses, significant modulation was evaluated using shorter windows—3 SD above baseline for at least 25 ms and 6 SD for at least 10 ms, reflecting the briefer duration of auditory compared to visual responses.

Significant modulation was defined by a *t-*test relative to baseline. All statistical procedures for neural data analysis were done using two-tailed tests unless otherwise specified. Mean values are reported with the ± standard deviation. The value of 0.01 was used as the alpha criterion for significance. If the sample size was small (*n* < 5), a Fisher’s exact test was used. All statistics were performed using MATLAB 2024b/2025a (MathWorks Inc; Natick, MA, USA).

Detection of visual responses was restricted to the first 150 ms after target presentation and the first 100 ms after the tone. The first peak of spike density functions was identified using MATLAB’s *findpeaks* function. The prominence of a peak measures how much the peak stands out due to its intrinsic height and its location relative to other peaks. A low, isolated peak can be more prominent than a higher peak that is surrounded by other peaks. A minimum peak prominence threshold of 3 spikes/s was determined through trial and error to balance sensitivity and specificity. If neurons had multiple peaks, we measured only the first, even if subsequent peaks were higher.

Within trials, periods of significant facilitation and suppression were detected using a Poisson spike train analysis (Legéndy and Salcman 1985; Hanes et al. 1995). Briefly, the algorithm determines how improbable it is that the number of action potentials within a specific time interval is a chance occurrence. This is achieved by comparing the observed number of spikes within a time interval to the number of spikes predicted by the Poisson distribution derived from the average discharge rate during the entire time period in which deviations from randomness are sought. While the algorithm can pick up multiple intervals with significant modulation, we only considered the first interval after target presentation.

Because monkeys exhibited different RTs across saccade directions, for any analysis that involved a comparison in neural activity between ipsilateral and contralateral targets, RTs were matched across same-direction saccades with 10-ms resolution. If multiple matching saccade RTs occurred, the trial with the closest timestamp in the session was selected.

To detect the pre-excitatory pause (PEP), we adapted method from Sato and Schall (2001), which quantifies the beginning of a transient dip in activity before the facilitatory target response. In a sliding window that was 40 ms wide, we determined whether successive spike density values over time were correlated according to a nonparametric Spearman rank-order test. Starting with the window centered on the minimum firing rate during the pre-excitatory pause, the window was moved backward in 1-ms steps until the test became nonsignificant (*P* > 0.01). The time of the center of the window producing the earliest significant positive correlation was defined as the time of the pre-excitatory pause.

Multiple post hoc Mann–Whitney two-way rank sum comparisons with Bonferroni correction were conducted to compare latencies between areas.

### Data analysis: biophysical neuron classification

**Figure 2:**
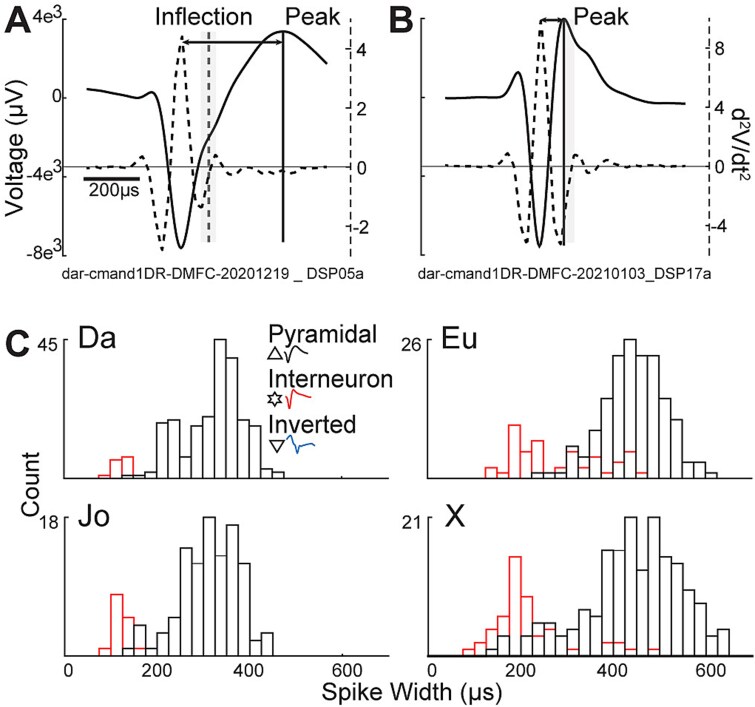
Spike waveform analysis. (A) Representative broad spike exhibiting an inflection (dashed vertical) before the peak of the action potential (solid vertical). Voltage (black) and its second derivative with respect to time (gray) are plotted. Spike width as the interval between the trough and the peak is highlighted by the arrow. (B) Representative narrow spike exhibiting no inflection before the peak of the action potential. (C) Distributions of spike widths measured for each monkey. Calibrated Hartigan’s dip test indicate multimodality for spike widths of all monkeys (Da dip = 0.086, *P* < 1e−4; Eu dip = 0.061, *P* < 1e−4; Jo dip = 0.071, *P* < 1e−4; X dip = 0.047, *P* < 1e−4). Black bins plot widths of units exhibiting the inflection. Red bins plot widths exhibiting no inflection. We also distinguished inverted units with the peak preceding the trough (not shown in histogram). The inset introduces symbols used in subsequent figures.

We sorted neurons based on the shape of their extracellular action potential (Fig. 2). In general, extracellular spike waveforms consist of a negative trough associated with fast sodium currents followed by a positive peak associated with slower potassium currents. Using trough-to-peak duration to distinguish narrow and broad extracellular spikes has been a common approach with the dip in the spike width histogram defining the two categories. Narrow-spiking waveforms are supposed to identify interneurons and broad-spiking, pyramidal neurons. However, this identification is not certain (Lemon et al. 2021).

The shape of extracellular spike waveforms is dictated by the unique expression of ion channels across neuron types (Gold et al. 2006; Anastassiou et al. 2015). Hence, we utilized another characteristic of the spike waveforms, an inflection between the trough and peak of the waveform observed in pyramidal neurons of the rat hippocampus (Csicsvari et al. 1999; Henze et al. 2000). The inflection arises in the transition from the Na^+^-dominant phase to the K^+^-dominant phase (Gold et al. 2006). Spike waveforms with the inflection can be simulated by a repolarization involving A- and C-type K^+^ currents, which possess different kinetics. The A-type K^+^ current is fast activating, quickly counteracting the depolarizing Na^+^ current, but also fast inactivating. In contrast, the C-type K^+^ current is both slower to activate and inactivate. The inflection occurs as the K^+^ phase slows when only the C-type K^+^ current continues the repolarization. The channels producing the A-type K^+^ current make a larger contribution to the conductance of hippocampal pyramidal neurons than to that of interneurons (Migliore et al. 2018). Previous work has shown that the presence of this inflection can distinguish pyramidal and interneurons in posterior parietal cortex (Thirunavukkarasu 2021; Thirunavukkarasu and Pare 2023).

First, we aligned spike waveforms for each unit to the global minimum of each waveform. Second, we calculated each neuron’s mean spike waveform and resampled it to a standardized 5-μs resolution using cubic spline interpolation. Spike width was measured as the interval between the minimum (trough) and maximum (peak) of this mean waveform. Third, we used the first temporal derivative of each neuron’s mean spike waveform to detect the presence of the abrupt bend occurring after the point of maximum slope (peak voltage change) and before the spike peak (Fig. 2A). This knee was identified as the interval between the minimum following the trough and the subsequent local maximum in the second derivative trace. The knee time was defined as the midpoint between these extrema and corresponds approximately to the time of the second positive-going zero-crossing, as derived from the second derivative of the voltage with respect to time. Critically, the knee is found exclusively in broad-spiking neurons and is absent in narrow-spiking neurons (Fig. 2B). Henceforth, neurons with a knee were identified putatively as pyramidal neurons and neurons without a knee, as interneurons.

### Data analysis: current-source density

We used the same methods reported in Godlove et al. (2014) and Herrera et al. (2022) to calculate the current-source density (CSD) from the average local field potential across channels. The CSD identifies local dendritic current flow within gray matter where neural ensembles with interconnected dendrites undergo simultaneous depolarization, enabling detection of current summation at the mesoscopic level (Nicholson and Freeman 1975; Riera et al. 2012). We computed the CSD signal using the spline-iCSD method (Pettersen et al. 2006) as implemented in the CSDplotter toolbox (https://github.com/espenhgn/CSDplotter) and applied custom MATLAB scripts. We defined a tissue conductivity of 0.4 S/m for gray matter, Gaussian smoothing kernel SD of 0.15 mm, and cylinder diameter of 3 mm. iCSD allows us to generate a spatially continuous CSD representation. We performed nearest-neighbor interpolation between electrode contacts to a density of 10 μm and applied Gaussian filtering (200 μm). This smoothing was essential because CSD values were averaged across recording sessions after aligning in depth as detailed below. Alignments of CSD across sessions were achieved with increments less than the 50-μm, 100-μm, or 150-μm interelectrode spacing. Prior to calculating CSD, the average local field potential was calculated as the mean local field potential across trials aligned on array onset then applying a baseline correction by subtracting the average activation during the 100-ms preceding array or tone onset.

### Data analysis: cortical depth assignment

Neurophysiological data were analyzed relative to cortical depth. Relative to the pial surface, we assigned the boundary L1 and L2 at 0.22 mm, that between L3 and L5 at 1 mm, and that between L5 and L6 at 1.25 mm (Matelli et al. 1991). For all monkeys, the depth of the electrode array relative to the pial surface and the gray–white matter boundary was assessed through the alignment of several independent physiological measures. This was approached as a two-step process.

**Figure 3:**
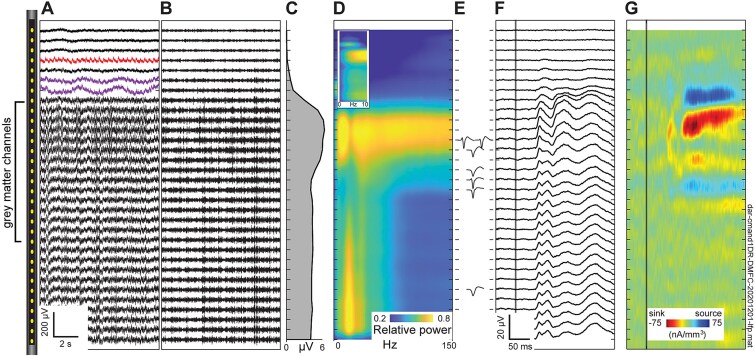
Laminar sampling. (A) Raw LFP sampled from channels spanning SEF. Artifacts from heartbeat (red) and respiration (purple) were observed in the channels outside the brain. (B) LFP bandpass filtered between 40 and 80 Hz to highlight gamma band power. (C) Magnitude of root mean square of LFP. (D) Plot of LFP spectral power across depth. The inset highlights the power at low frequencies in channels outside the brain derived from ECG. (E) Spike waveforms isolated on channels across depth. (F) Mean visually evoked local field potential. (G) Visually evoked CSD.

In the first step, contiguous channels carrying signals consistent with arising in the cortical gray matter were identified. This coarsely aligned the depths of contacts across sessions at the resolution of the interelectrode spacing. Several features were incorporated to determine the depth of the pial boundary (Fig. 3). First, in certain sessions, an electrocardiogram signal was observed on superficial channels which indicated where the electrode was in contact with either the dura mater or epidural saline in the recording chamber and thus could not be in the cortical gray matter. Second, contacts in the upper cortical areas were distinguished by elevated power in the gamma frequency range (40 to 80 Hz) (Maier et al. 2010; Xing et al. 2012; Smith and Sommer 2013; Godlove et al. 2014; Ninomiya et al. 2015; Mendoza-Halliday et al. 2023). This elevated gamma power commonly coincided with a larger magnitude of the root-mean-squared amplitude of the LFP. Third, the CSD derived from visually evoked and acoustic-evoked LFP exhibited consistent source/sink structures consistent with previous observations (Godlove et al. 2014). Fourth, the density of isolated spiking units within sessions indicated which channels were in the cortical gray matter. Converging evidence from these different lines of evidence provided very good estimates of consistent depth across sessions at the resolution of the spacing of the electrode contacts.

**Figure 4:**
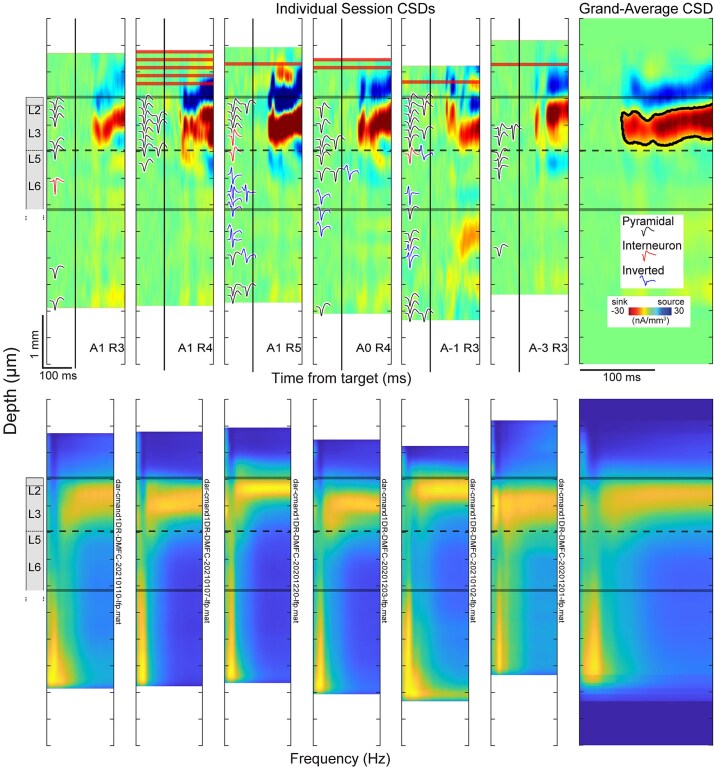
Aligning samples in depth. (Top) CSD evoked by visual target (thick vertical line) for 6 penetrations in right hemisphere of Da with grand-average CSD on right. Spike waveforms isolated on contacts at each depth are superimposed. Black waveforms mark spikes with inflection in repolarization (pyramidal neurons). Red waveforms mark spikes without inflection (interneurons). Blue waveforms are inverted or otherwise unusual. Red horizontal lines mark channels with ECG. Thick horizontal lines indicate pial and white matter boundaries. (Bottom) Laminar distribution of LFP spectral power in each penetration and their average.

The second step applied an automated depth alignment procedure to fine tune alignments at a higher resolution than the interelectrode spacing (Godlove et al. 2014) (Fig. 4). To calculate CSDs for alignment, only local field potential channels within the putative gray matter were included, defined from the first identified gray matter channel to a depth of 2,100 μm determined by electrode spacing (50 μm, 100 μm, 150 μm). The value 2,100 μm was selected as a common multiple between all electrode spacings and approximates the laminar depth of SEF (Matelli et al. 1989). However, the physical span of the recording probe was shorter than this distance typically in sessions with higher-resolution sampling; 32 channels at 50 μm spans only 1.6 mm. In other sessions, the cortex was deeper than the final electrode contact. In these cases, the CSD matrices were zero-padded to fill the remaining spatial points. Single-session CSD matrices were normalized by their global standard deviation and thresholded such that values < 1 SD in magnitude were set to zero to emphasize prominent current sources and sinks (Fig. S1A).

The alignment procedure used an iterative refinement process, terminating upon convergence up to a maximum of 200 iterations. At each iteration, a grand-average CSD was computed from the current alignment of all sessions. For each session, similarity to this grand average was quantified by calculating spatial cross-correlations at each time point and averaging across time to yield a single agreement metric (Fig. S1B).

The algorithm then sought to maximize the agreement metric by systematically shifting the depth of displacing each session’s matrix within a bounded range. For each candidate shift, Pearson cross-correlation with the grand average was recomputed, and the shift yielding the highest similarity was applied. To maintain anatomical plausibility, each session tracked its cumulative displacement, which could not exceed ±15 samples (10 μm/sample) across all iterations. As sessions became progressively better aligned to the evolving grand average, the mean representation stabilized, and shift magnitudes diminished. Convergence was achieved when no additional shifts improved cross-correlation, indicating that optimal alignment had been reached. This required typically 5 and never more than 10 shifts.

The location of the pial surface was estimated from the grand-average CSD. After computing CSDs for each session and normalizing them, a grand mean was generated across all sessions. A statistical threshold was applied to this average to identify continuous patches of significant current flow near the superficial contacts. The shallowest depth of these patches was taken as the initial estimate of the pia. Because the spline-iCSD method and electrode referencing can bias the apparent depth of the surface, a small manual correction was applied. For monkey Jo, the superficial estimate was adjusted an additional fine offset of 0.3 mm. For the other monkeys, a uniform 0.15 mm correction was applied. From this starting point, the gray matter was defined as spanning a 2.1-mm band, and all subsequent CSD and PSD data were referenced to this boundary.

As converging evidence, we quantified the depth distribution of isolated units across monkeys by constructing normalized depth histograms. Unit depths were binned at 0.15-mm intervals, and distributions were computed separately for each monkey. To facilitate cross-animal comparison, histograms were aligned by applying small depth shifts that minimized the squared error between each monkey’s distribution and the pooled reference distribution of depths across all animals (Fig. S2). We applied the isolated unit density instead of current-source density (CSD) for cross-monkey alignment. Unlike CSD, which is sensitive to electrode angle, referencing, and task-dependent synaptic activity, unit density provides a stable measure of laminar composition that is less affected by recording idiosyncrasies. Final session alignments for all animals are provided in Figs. S3 and S4.

## Results

We acquired recordings in 88 sessions from 4 macaques (Da: 32, Eu: 12, Jo: 27, X: 17) performing the saccade-countermanding task. Some sessions were omitted from further analysis due to faulty electrodes with poor signal-to-noise ratio or dead channels (five in monkey Da, one in monkey Jo) or too large signal artifacts potentially due to insufficient grounding (three in monkey Jo, one in monkey X). We report SEF data from 65 sessions (Da: 24 sessions, Eu: 12 sessions, Jo: 13 sessions, X: 16 sessions). From contacts in the ~ 2 mm of cortical depth of SEF, we isolated 869 single units (Da: 247, Eu: 222, Jo: 125, X: 275). From each session, we analyzed exclusively no-stop trials which the monkeys performed correctly to avoid the influence of evaluative extraretinal error or conflict signals. The laminar description of these trial types will be reported separately. In each session, we analyzed ~ 140 to ~ 1,020 trials (Da: 570 to 1,022 trials, Eu: 136 to 484 trials, Jo: 624 to 878 trials, 186 to 918 trials), split evenly between ipsilateral and contralateral targets and matched by saccade latency as described earlier. Saccade latencies varied between animals (median RT Da: 303 to 394 ms, Eu: 248 to 304 ms, Jo: 273 to 345 ms, X: 217 to 259 ms; minimum RT Da: 164 to 209 ms, Eu: 67 to 167 ms, Jo: 151 to 218 ms, X: 95 to 181 ms).

By design, we sought information about the boundaries of SEF by sampling surrounding areas. From penetrations medial to SEF, we isolated 76 units. These were found in monkey Da (*n* = 53) in two penetrations 32 and 33 mm anterior to the interaural line and 3 mm lateral to the midline and in monkey Jo (*n* = 23) in one penetration 28 mm anterior to the interaural line and −2 mm lateral to the midline. Based on finding isolated single units from contacts contiguous over more than 2 mm and the structure of CSD and spectrolaminar plots differing markedly from those of the perpendicular penetrations in SEF, we surmise that these penetrations sampled from pre-SMA in the medial wall. From a penetration lateral to SEF in monkey Da, 32 mm anterior and 6 mm lateral, we isolated 22 units across ~ 2 mm of cortical depth, all of which responded only to the auditory tone. We surmise that this penetration was in the premotor ear–eye field (Lucchetti et al. 2008). From four sessions sampled, the prefrontal area rostral to SEF in monkey Jo 35 to 36 mm rostral to the interaural line and 4 to 5 mm from the midline, we isolated 23 units across ~ 2 mm of cortical depth. These are in medial area 8b or area 9. We did not sample caudal to SEF because the upper limb and face representation in SMA has been described (eg Schall 1991a). Ventrally, in several sessions, deeper contacts sampled neurons in the dorsal bank of the cingulate sulcus. These data will be reported separately.

### Putative neuron type

We sorted neurons based on the shape of their extracellular action potential. Previous studies have used the interval from trough to peak to distinguish biophysical neuron types with a value of 250 μs differentiating narrow from broad spikes (Cohen et al. 2009; Thiele et al. 2016). As in other cortical areas, the distribution of spike widths from SEF for each monkey was multimodal (calibrated Hartigan’s dip test, *P* < 0.001), but we observed notable differences in spike widths recorded between subjects (Fig. 2C). To address the uncertainties inherent in this method, we utilized another characteristic of the spike waveforms, an inflection between the trough and peak of the waveform that distinguishes pyramidal and interneurons (Csicsvari et al. 1999; Henze et al. 2000; Thirunavukkarasu 2021; Thirunavukkarasu and Pare 2023). We identified 135 putative interneurons (Da: 17, Eu: 49, Jo: 15, X: 54) and 658 putative pyramidal neurons (Da: 210, Eu: 165, Jo: 105, X: 178). The 1-to-4.9 ratio of interneurons to pyramidal neurons is smaller but similar to the 1- to -4 ratio reported previously (Dzaja et al. 2014; Ardid et al. 2015). We also identified 76 neurons with inverted spike waveforms (Da: 20, Eu: 8, Jo: 5, X: 43) generally observed when sampling from deeper channels and attributed to axonal recordings (Gold et al. 2006; Barry 2015).

### Functional properties of visual and auditory responses in the supplementary eye field

**Figure 5:**
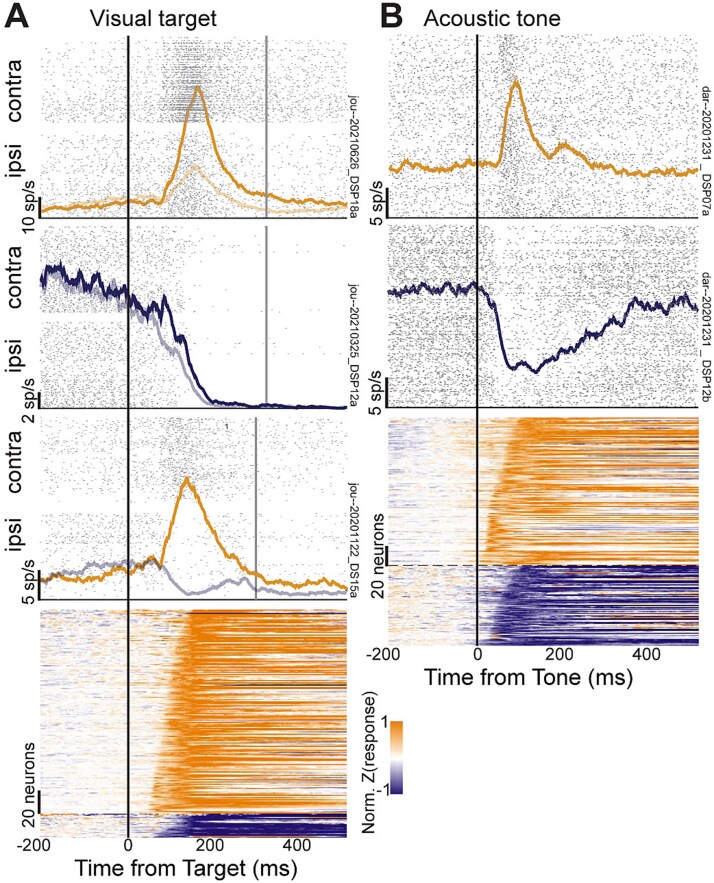
Representative visual and auditory response patterns. (A) Raster and average spike density of representative visually responsive neurons in response to contralateral (darker) and ipsilateral (lighter) targets. The *x*-axis corresponds to 0 spikes/s. Neurons with facilitation (top) and suppression (second), or both (bottom) are shown. Median RT is marked by vertical gray line. Fourth panel shows color map raster of average spike density for hemifield with the strongest modulation for all facilitated (orange) and suppressed (blue) neurons ordered by visual latency. (B) Representative responses to acoustic tone.

To identify visually responsive neurons, we examined the modulation immediately after the peripheral target was presented. We focused on no-stop trials, in which monkeys earned reward for shifting gaze to the target. Neurons were categorized based on the sign of visual response (Fig. 5). The counts reported below are summarized in Table 1.

**Table 1:** Distribution of neuron types by cortical area and subject.

Cortical area	Da	Eu	Jo	X	Total
SEF					
Total sample	247	222	125	275	869
Pyramidal	210	165	105	178	658
Interneuron	17	49	15	54	135
Inverted	20	8	5	43	76
Visual facilitated	45	65	39	30	179
Meet single-trial threshold	17	44	28	7	96
Contralateral strongest	7	16	19	0	42
Ipsilateral strongest	4	17	4	7	32
Bilateral	6	11	5	0	22
Transient	3	2	2	0	7
Sustained	14	42	26	7	89
Visual suppressed	9	2	8	2	21
Meet single-trial threshold	9	2	7	1	19
Contralateral strongest	4	0	2	0	6
Ipsilateral strongest	4	4	4	1	13
Bilateral	1	0	1	0	2
Auditory facilitated	51	37	29	32	149
Meet single-trial threshold	17	25	0	5	47
Auditory suppressed	33	11	11	26	81
Meet single-trial threshold	15	7	3	7	32
Bimodal	8	9	1	2	20
Area 8b/9 rostral to SEF					
Total sample	…	…	23	…	23
Visual facilitated	…	…	3	…	3
Meet single-trial threshold	…	…	3	…	3
Visual suppressed	…	…	0	…	0
Meet single-trial threshold	…	…	0	…	0
Auditory facilitated	…	…	2	…	2
Meet single-trial threshold	…	…	2	…	2
Auditory suppressed	…	…	1	…	1
Meet single-trial threshold	…	…	0	…	0
Bimodal	…	…	0	…	0
Premotor ear–eye field lateral to SEF					
Total sample	22	…	…	…	22
Visual facilitated	0	…	…	…	0
Meet single-trial threshold	0	…	…	…	0
Visual suppressed	0	…	…	…	0
Meet single-trial threshold	0	…	…	…	0
Auditory facilitated	20	…	…	…	20
Meet single-trial threshold	20	…	…	…	20
Auditory suppressed	2	…	…	…	2
Meet single-trial threshold	2	…	…	…	2
Bimodal	0	…	…	…	0
Pre-SMA medial to SEF					
Total sample	53	…	23	…	76
Visual facilitated	4	…	6	…	10
Meet single-trial threshold	2	…	1	…	3
Visual suppressed	2	…	0	…	2
Meet single-trial threshold	2	…	0	…	2
Auditory facilitated	2	…	0	…	2
Meet single-trial threshold	2	…	0	…	2
Auditory suppressed	1	…	1	…	2
Meet single-trial threshold	0	…	0	…	0
Bimodal	0	…	0	…	0

In SEF, we observed 211 (~24% of total sample) neurons with significant modulation from baseline following the presentation of a visual target in either one or both hemifields. Of these, 179 (~85%) produced facilitated firing rates, and 21 (~10%), suppressed. An additional 11 (~5%) units exhibited facilitation when the target appeared in one hemifield and suppression when the target appeared in the other. From area 8b/9, we observed three responsive neurons (~13% of total sample) with three producing facilitation and none with suppression. In a penetration lateral to SEF, no neurons with visual responses were found. In medial samples, we observed ~ 16% responsive neurons with 10 facilitated and 2 suppressed.

**Figure 6:**
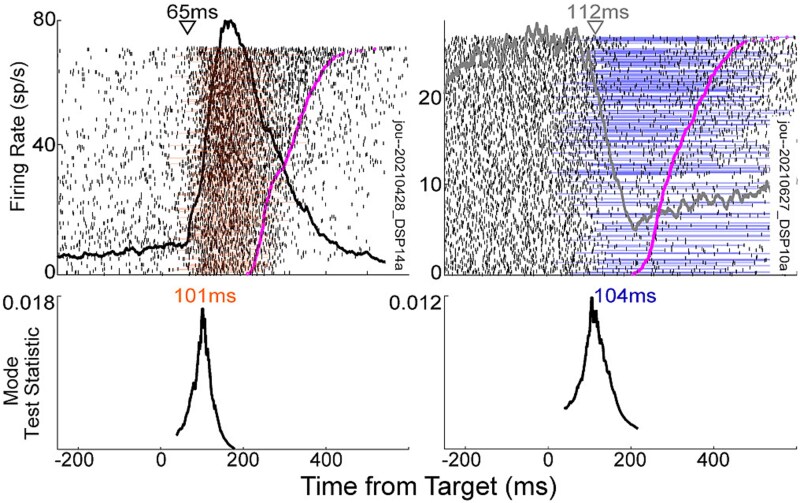
Visual response measures. *Top*: Raster and average spike density of representative visually responsive facilitated (left) and suppressed (right) neurons exhibiting consistent trial-by-trial modulation detected by a Poisson single-trial modulation detection algorithm. SDFs and rasters are from trials from the hemifield with a dominant response and are ordered by saccade latency (magenta). Color conventions follow Fig. 3. Significant modulation onset calculated from the SDF are marked with inverted triangles. Horizontal colored bars show significant periods of increased firing for the facilitated neuron (orange bars) and decreased firing for the suppressed neuron (blue bars). *Bottom*: Mode estimation for beginning of the trial-wise modulation. Peak of function is modal latency. Latencies reported are the midpoint between both methods of modulation onset detection.

Although many neurons showed some modulation after target appearance, we restricted further analyses to units with clear, stimulus-locked either facilitated or suppressed visual responses. We verified that post-target activity was synchronized with target presentation and dissociated from saccade production using a Poisson burst-detection algorithm (Hanes and Schall 1995) (Fig. 6). To be included in further analyses, neurons needed to exhibit a significant period of visual modulation in at least 30% of trials and exhibit visual activation before the earliest saccade. Of the 179 neurons exhibiting facilitated responses, 96 met these criteria for salient visual activity. Similarly, suppression onset was identified by adapting the Poisson spike train analysis to detect periods with significantly fewer spikes than expected by chance on a trial-by-trial basis (Pouget et al. 2005; Royal et al. 2006). Because the Poisson analysis is less sensitive to decreases in firing, particularly when baseline rates are low, a more relaxed criterion was applied, requiring at least three trials to exhibit a significant period of suppression. Of the 21 neurons with suppressed responses, 19 met these criteria.

Among neurons sampled in area 8b/9 rostral to SEF, all met the criterion for facilitation (*n* = 3) and no suppressed neurons were found. Among neurons sampled lateral to SEF, no visually modulated neurons were found. Among penetrations medial to SEF, 3 of the 10 neurons met the criterion for facilitated modulation, and 1 of the 2 neurons met the criterion for suppressed modulation. All of these visually responsive neurons were encountered in the most dorsal 2.6 mm.

To identify auditory responsive neurons, we examined activity after presentation of the auditory tone delivered 600 (X, Eu) or 700 (Da, Jo) ms after saccades to the target. The counts reported below are also summarized in Table 1. In SEF, we observed 230 neurons (~26% of total sample) with significant modulation from baseline, with 149 (~65% of auditory neurons) facilitated responses and 81 (~35%) suppressed (Fig. 5). From area 8b/9, we observed three neurons (~13% of total sample) with significant modulation from baseline, where two neurons produced a significant increase in firing rates and one neuron had a significant decrease in firing rates. Penetrations lateral to SEF in Da encountered a high density of auditory responsive neurons where all 22 neurons recorded from this site exhibited an auditory response with 20 exhibiting a facilitated and 2 exhibiting a suppressed response. In the most medial contacts, we observed a few modulated neurons, 2 producing a significant increase in firing and 2 producing a significant decrease in firing rate.

We restricted further analyses to units with clear, stimulus-locked auditory responses. Post-tone activity was verified to be consistent across trials using the same Poisson burst-detection algorithm. Neurons were required to exhibit a significant period of auditory activation in at least 15% of trials. We applied a more liberal threshold because Poisson burst windows were briefer in response to auditory stimuli in comparison to visual stimuli. In SEF, 47 of the original 149 neurons with facilitated responses met these criteria. Suppression onset was similarly identified using the adapted Poisson analysis, with relaxed thresholds for decreases in firing. A minimum of 10% of trials with significant suppression was required. In total, 32 neurons with suppressed responses met these criteria. Applying these criteria to area 8b/9, two neurons met the criterion for facilitated modulation and none for suppressed modulation. Applying these criteria to lateral penetrations, all neurons met the criterion for facilitated modulation or suppressed modulation. Applying to medial penetrations, 2 neurons met the criterion for facilitated modulation, and no neurons met the criterion for suppressed modulation.

Among all visual or auditory neurons in SEF, we found 66 (~31% of visually responsive and ~ 29% of auditory responsive) that were bimodal; 41 were bimodally facilitated, 4 neurons were bimodally suppressed, and 21 were facilitated to one modality and suppressed to the other. Among visual or auditory neurons which were found using the Poisson analysis in SEF, we found 20 (~17% of visually responsive and ~ 25% of auditory responsive) that were bimodal; 11 were bimodally facilitated, 2 neurons were bimodally suppressed, and 7 were facilitated to one modality and suppressed to the other.

#### Hemifield preferences of visual responses

**Figure 7:**
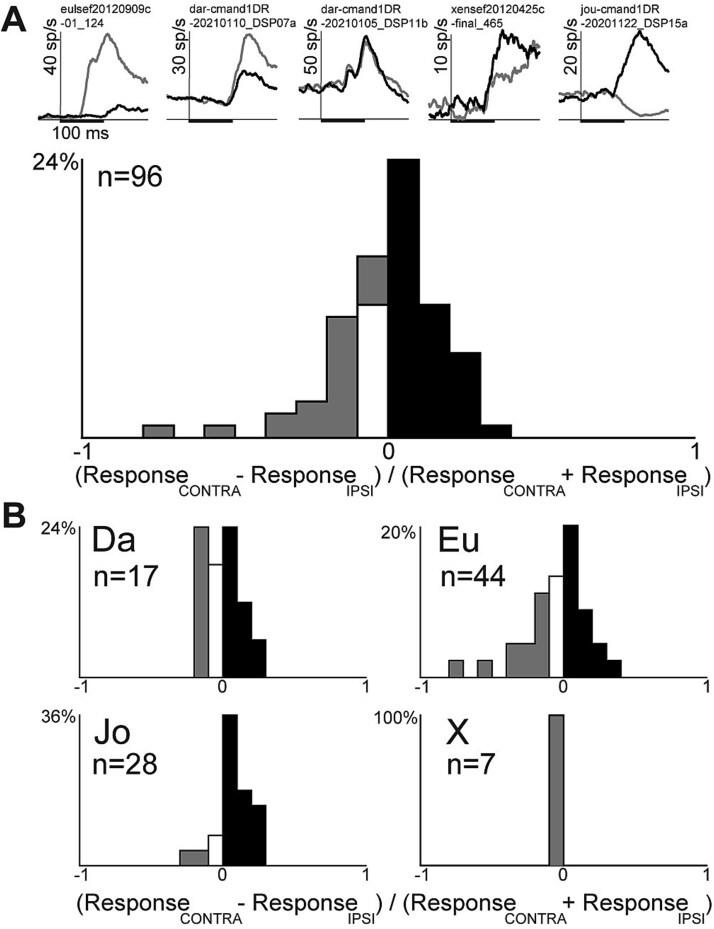
Hemifield sensitivity. (A) Distribution of contrast ratio of facilitated responses to contra- and ipsilateral targets. Stronger contralateral responses have positive values. Neurons with statistically significant stronger responses to contra- (black) or ipsilateral targets (gray) are distinguished. Spike density functions of representative neurons are shown above with responses to contra- (black) and ipsilateral (gray) targets distinguished. (B) Distributions for each monkey.

Targets appeared at 12° eccentricity on the horizontal meridian in either the contra- or ipsilateral visual field. We quantified the laterality of facilitated visual responses by calculating the contrast ratio: (Response _CONTRA_ − Response _IPSI_)/(Response _CONTRA_ + Response _IPSI_). Positive values represent stronger facilitated responses to contralateral targets, and negative, to ipsilateral targets (Fig. 7). Based on neuron-wise two-sample, two-tail *t-*tests, significantly stronger facilitation for the contralateral target was found for 42 SEF neurons (31 pyramidal, 11 interneuron, 0 inverted), significantly stronger ipsilateral for 32 neurons (19 pyramidal, 12 interneuron, 1 inverted), and indistinguishable for 22 neurons (18 pyramidal, 4 interneuron, 0 inverted). In monkey X, we found a curious numerically small but statistically significant dominance of responses to targets in the ipsilateral visual field. The spike density function calculated from the hemifield with the strongest response was used for all further analyses. For suppressed responses, the negative value of the contrast ratio was used, so positive (negative) values represent stronger suppression for contralateral (ipsilateral) targets. Significantly stronger facilitation for the contralateral target was found for 6 SEF neurons (all pyramidal), significantly stronger ipsilateral for 11 neurons (9 pyramidal, 2 interneuron, 0 inverted), and indistinguishable for 2 neurons (both pyramidal).

#### Transient and sustained visual responses

**Figure 8:**
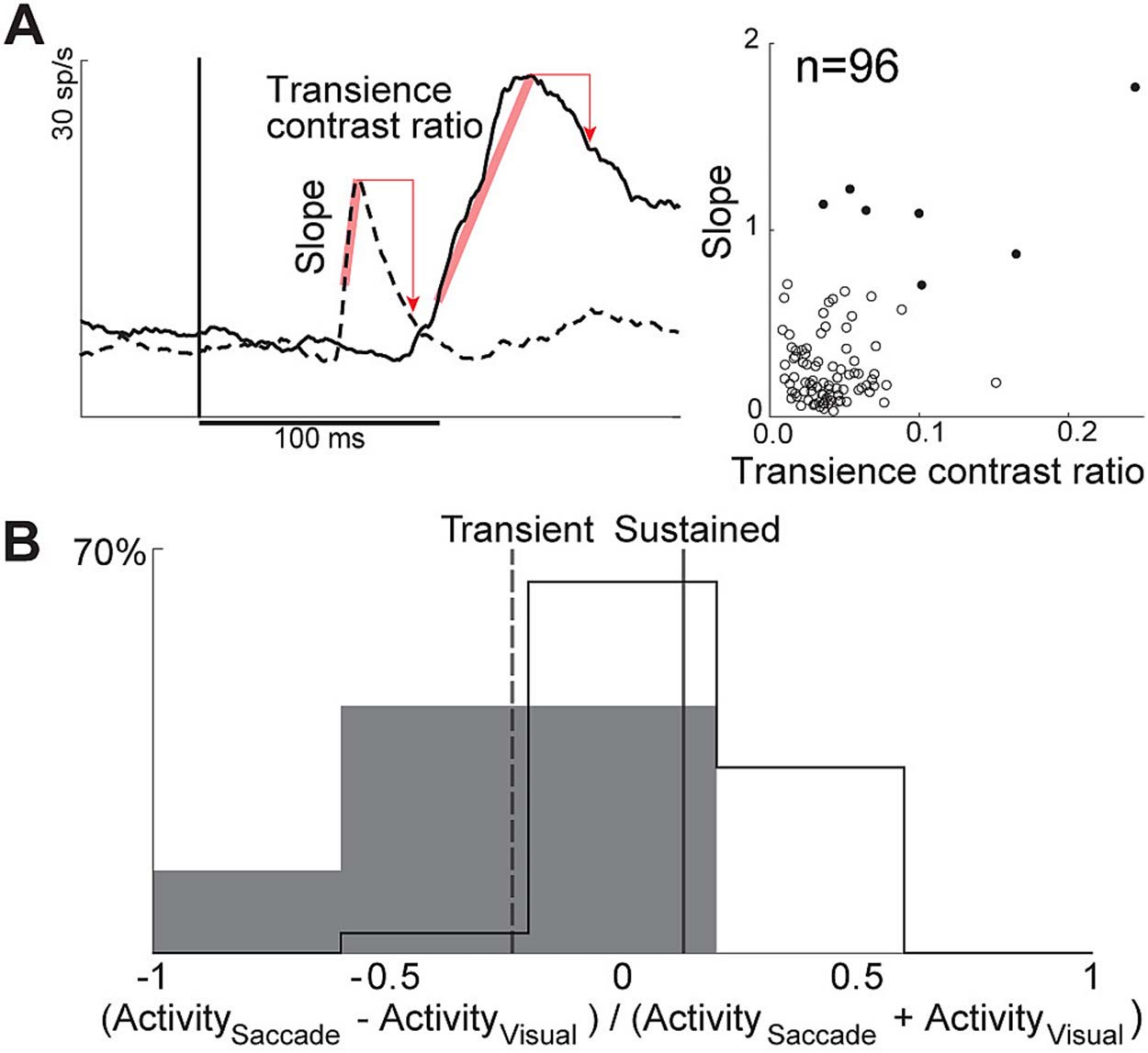
Transient and sustained responses. (A) Left, illustration of measurements obtained on spike density functions of a transient (dashed) and a sustained (solid) neuron. Two attributes were measured. The first was the slope of the spike density function from the latency of the visual response to the peak of the response (light red). The second was a transience contrast ratio between the peak spike rate and the spike rate 25 ms after the peak (dark red arrow). Right, scatter plot of slope relative to transience contrast ratio; larger values index more transient responses. Neurons with transient visual responses (filled) were separated from neurons with sustained visual responses (open) using k-medoids clustering. (B) Histogram of visuomotor contrast ratio for transient (filled) and sustained (open) neurons. Vertical lines show mean values for transient (dashed) and sustained (solid) responses.

Different visual response profiles elicited by the target were observed. Facilitated neurons exhibited either sustained or transient responses (eg Cleland et al. 1971; Cleland and Levick 1974) (Fig. 8A). To distinguish between transient and sustained visual responses, we measured the slope from the initiation of the response to the peak spike rate and a transience contrast ratio derived from the peak spike rate response and the mean spike rate 25 ms after the peak (Lisberger and Movshon 1999; Osborne et al. 2004). Applying the MATLAB k-medoids algorithm (Statistics and Machine Learning Toolbox), the distribution of these paired values was sorted into two clusters. Most were sustained, but seven (~7%) had transient responses.

We also quantified the balance of visually evoked and saccade-related activity between transient and sustained responses by measuring discharge rates in the intervals 0 to 150 ms after visual target presentation and ±50 ms relative to saccade initiation. Trials with overlapping visual and motor intervals were omitted. Values < 0 represent stronger visual activity, and values > 0, stronger saccade-related activity. Typically, neurons with ratios less than −0.4 are categorized as visual while values greater than 0.4 are saccadic, with the remainder identified as visuomotor (Bruce and Goldberg 1985; Lawrence et al. 2005). Neurons with sustained responses were nearly all visuomotor (81/89, ~ 91% of sustained neurons, mean ratio = 0.132 ± 0.192) (Fig. 8B). Of the sustained neurons, 1 was visual, falling below the minimum threshold of −0.4, while 7 exceeded the maximum threshold of 0.4. Of the transient neurons, 2 were visual with ratios less than −0.4. The distributions of visuomotor ratios were significantly different between transient and sustained responses (two-sample *t*-test, *t*(94) = 4.720, *P* < 0.001). Of the transient neurons, 5 were identified as visuomotor, and none identified as strictly saccadic. Most sustained neurons were pyramidal (64 pyramidal, 24 interneuron, 1 inverted), while transient were more balanced (4 pyramidal, 3 interneuron, 0 inverted). The likelihood of responding best to contra- or ipsilateral targets was balanced for both transient (4 contralateral: 2 ipsilateral: 1 binocular) and sustained (38 contralateral: 30 ipsilateral: 21 binocular) neurons. No neurons in area 8b/9 were transient.

#### Pre-excitatory pause and pause-rebound neurons

**Figure 9:**
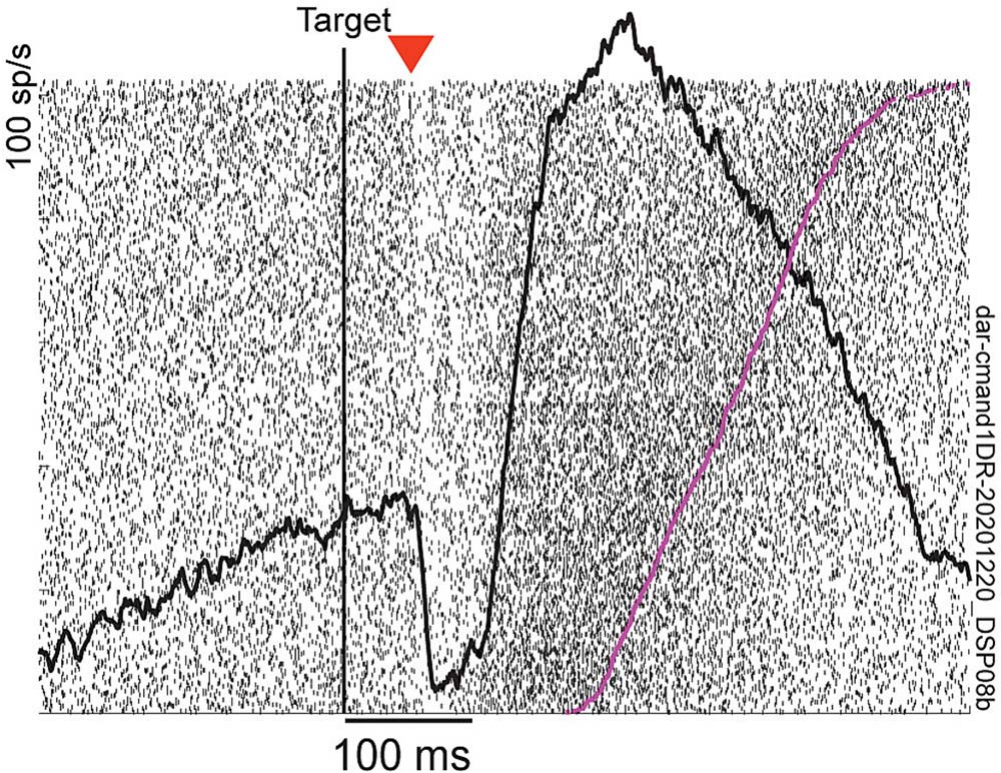
Pre-excitatory pause. Raster and average spike density for a representative neuron. Conventions as in Fig. 6. Red triangle indicates beginning of PEP.

Some of the neurons with facilitated sustained responses exhibited a short pause before the facilitation (Fig. 9). This pre-excitatory pause was reported in frontal eye field and other visual areas (Sato and Schall 2001, Shinn et al. 2022). We observed 10 neurons for which spike rate before the facilitation was transiently reduced more than 3 SD below the baseline. The pause began 12 ± 30.7 ms after target presentation, with a median of 14 ms. The transient dip in activity was detected at 46 ± 22.6 ms, with a subsequent significant rise detected at 58 ± 20.6 ms and facilitatory activation at 87 ± 24.0 ms. Like FEF, this average is so small because the pre-excitatory pause of some neurons was measured before target presentation (range: −28 ms to 53 ms). The duration of the pause was 75 ± 19.7 ms. PEP was not detected rostral or medial to SEF.

**Figure 10:**
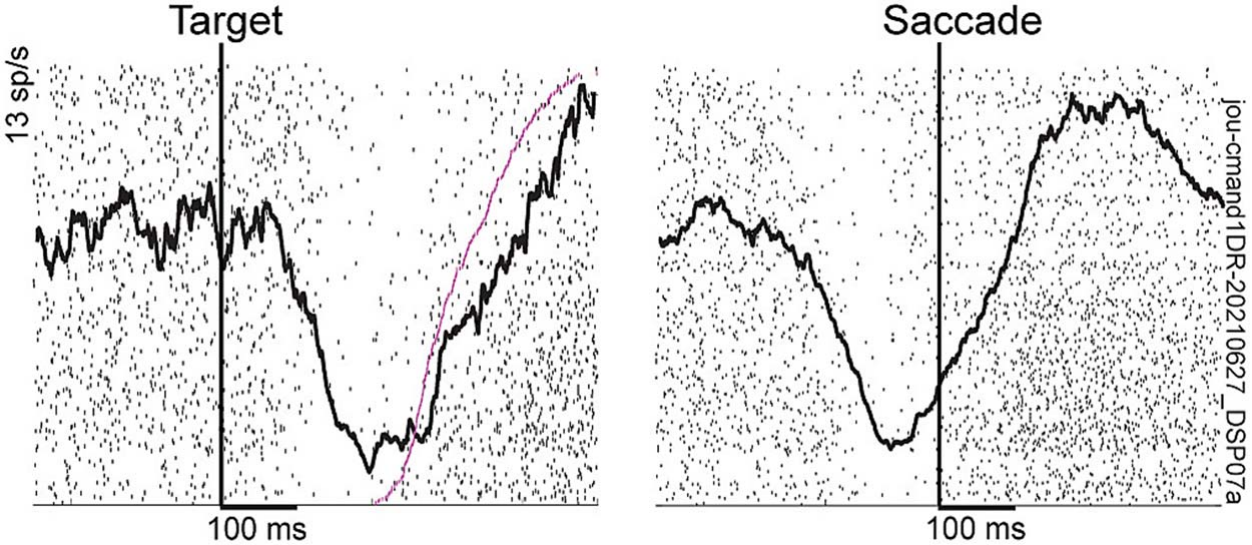
Pause-rebound. Raster and average spike density aligned on target presentation (left) and gaze shift (right) for a representative neuron. Conventions as in Fig. 6.

Among the suppressed sustained responses, we observed other neurons with a biphasic pattern of suppression until a burst of activation synchronized on initiation of the saccade (Fig. 10, also see Fig. S5). These were referred to as pause-rebound neurons (Schall 1991a) and may be identified as fixation neurons (Schlag and Schlag-Rey 1987; Heinen 1995; Bon and Lucchetti 1997). The suppression observed among these neurons was sustained compared to the transient pre-excitatory pause. In SEF, we observed one neuron with a transient suppressed response to the visual target and a transient facilitated response upon saccade production and four neurons with more sustained responses to these events. The mean onset of suppression was 72 ± 17.6 ms. Pause-rebound neurons were not detected rostral or medial to SEF.

#### Visual latencies (facilitated)

Latencies of visual and auditory responses were measured two ways. The first was the instant when the average spike density function departed from a baseline level. Based on this approach, the latencies of the facilitated visual responses ranged from 44 to 145 ms with an overall mean ± SD of 82 ± 22.6 ms. The first quartile of neurons was active within 60 ms, one half of the neurons had latencies under 82 ms, and the third quartile was active within 96 ms. The second measurement was the mode of the beginning of modulation detected by the Poisson single-trial algorithm (Thompson et al. 1996). Based on this approach, the latencies of the facilitated responses ranged from 20 to 168 ms with an overall mean of 90 ± 30.0 ms. The first quartile of neurons was active by 66 ms, one half of the neurons had latencies under 92 ms, and the third quartile was active within 108 ms Because the latencies measured by both methods were significantly correlated (Pearson correlation: *r* = 0.43, *P* < 0.001), and the average latencies calculated from both distributions were only marginally different (Mann–Whitney *U* (96,96) = 8,415.50, *P* = 0.03), the latency for each neuron was taken as the midpoint of the value obtained from the two measurement methods. Thus, the latencies of facilitated visual responses ranged from 45 to 141 ms with an overall mean of 86 ± 22 ms. The first quartile of neurons was active within 70 ms, one half of the neurons had latencies under 88 ms, and the third quartile was active within 98 ms. The facilitated visual response latencies occurred well before saccades with the average value of the earliest saccade across sessions being 166 ± 35 ms (*U*(96,96) = 5,049.0, *P* < 0.001).

We noted signification variation in the latencies of facilitated visual responses for neurons responding to the target in the ipsilateral (78 ± 21.1 ms), contralateral (87 ± 22.5 ms), or both (95 ± 19.5 ms) hemifields (Kruskal–Wallis, *H*(2) = 7.2190, *P* = 0.03). The latencies of neurons with ipsilateral responses were significantly shorter than those with bilateral responses (*U*(32,22) = 208.0, *P* = 0.01).

Visual latency of facilitated responses varied with biophysical properties of the neurons. Because there was only one inverted neuron, it was excluded from this analysis. The visual response latency of pyramidal neurons (90 ± 21.5 ms) was significantly later than that of interneurons (76 ± 20.1 ms) (*U*(68,27) = 957.0, *P* < 0.01). The visual latency of facilitated responses also varied significantly between transient and sustained responses. The visual response latency of neurons with transient responses (56 ± 6.5 ms) was significantly earlier than that of neurons with sustained responses (88 ± 21 ms) (*U*(89,7) = 84.5, *P* < 0.001).

**Figure 11:**
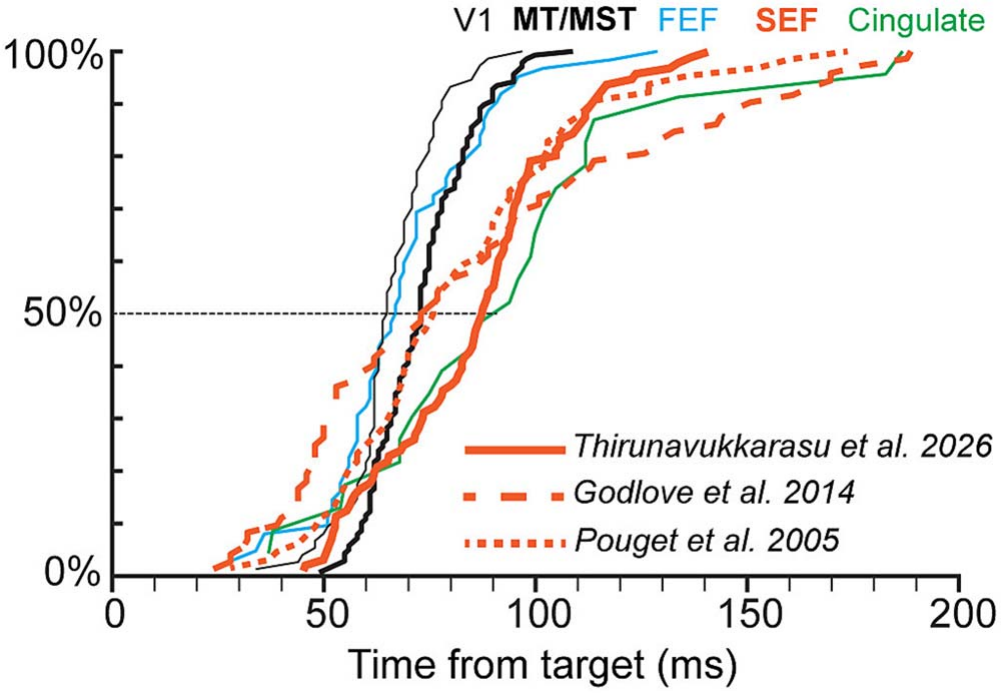
Comparing visual latencies. Cumulative distributions of visual responses reported previously in V1 (thin black), MT and MST (thick black), and FEF (blue) (Schmolesky et al. 1998) and in SEF (orange) measured here. For comparison are included previous measurements from SEF (dotted orange) and cingulate cortex (green) of other monkeys during the same task (Pouget et al. 2005) and in response to full-screen bright flashes (dashed orange, Godlove et al. 2014).

The latencies of visually facilitated neurons were indistinguishable from those previously reported in SEF sampled during a countermanding task (Fig. 11) (median = 76 ms; Mann–Whitney *U*(64,96) = 2,614.5, *P* = 0.11) (Pouget et al. 2005) or in response to full-field flashes (median = 74 ms; *U*(72, 96) = 2,932.5, *P* = 0.09) (Godlove et al. 2014). Significant variation in latency compared to other visual cortical areas previously reported (Schmolesky et al. 1998; Pouget et al. 2005) was confirmed by a Kruskal–Wallis one-way ANOVA on ranks (*H*(4,393) = 59.91, *P* < 0.001) (Fig. 11). In comparison to latencies measured in other cortical areas, the present values were not different from those measured in the cingulate cortex (median = 94 ms; *U*(23,96) = 994.5, *P* = 0.46) (Pouget et al. 2005). Meanwhile, the latencies of visual responses in SEF were significantly later than those reported by Schmolesky et al. (1998) in V1 (median = 65 ms; *U*(74,96) = 5,483.0, *P* < 0.001), MT and MST (median = 73 ms; *U*(138,94) = 9,115.0, *P* < 0.001), and FEF (median = 73 ms; *U*(62,96) = 4,291.0, *P* < 0.001).

#### Visual latencies (suppressed)

The latencies of the suppressed visual responses ranged from 52 to 114 ms with an overall mean of 79 ± 17.9 ms. The first quartile of neurons was active at 71 ms, one half of the neurons had latencies under 77 ms, and third quartile was active within 86 ms. Visual latency of suppressed responses did not vary with biophysical properties of the neurons (pyramidal neuron onset: 79 ± 18.8 ms, interneuron: 80 ± 10.8 ms; *U*(17,2) = 21.0, *P* = 0.95).

#### Auditory latencies (facilitated)

The latencies of the facilitated auditory responses ranged from 12 to 95 ms with an overall mean of 58 ± 24.2 ms. The first quartile of neurons was active at 45 ms, one half of the neurons had latencies under 59 ms, and third quartile was active within 81 ms. The latency of facilitated auditory responses did not vary with biophysical properties of the neurons. The auditory response latency of pyramidal neurons (60 ± 24.2 ms) was not different than that of interneurons (50 ± 26.1 ms) (*U*(34,10) = 183.5, *P* = 0.25). Only three inverted neurons were identified, so they were excluded from this analysis.

#### Auditory latencies (suppressed)

The latencies of the suppressed auditory responses ranged from 11 to 85 ms with an overall mean of 36 ± 14.6. The first quartile of neurons was active at 25 ms, one half of the neurons had latencies under 33 ms, and third quartile was active within 42 ms. The latency of suppressed auditory responses did vary with biophysical properties. The auditory response latency of pyramidal neurons (33 ± 10.8 ms) was significantly earlier than that of interneurons (57 ± 17.1 ms) (*U*(25,5) = 131.0, *P* < 0.01). Only two inverted neurons were identified.

### Functional architecture of visual responses in the supplementary eye field

#### Areal topography

**Figure 12:**
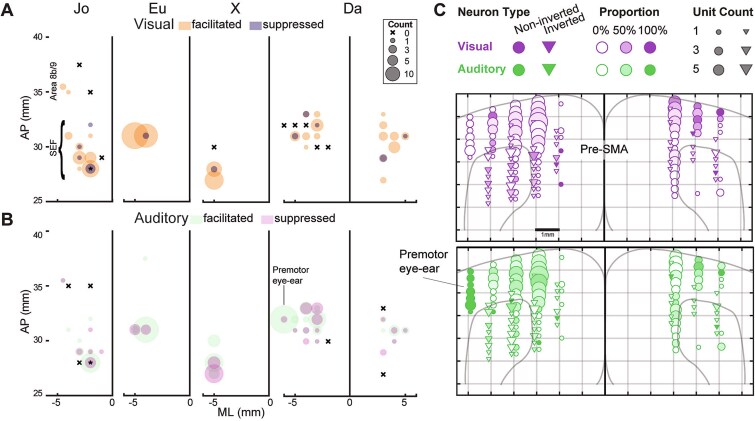
Localization of neurons. (A) Stereotaxic coordinates of facilitated (orange) and suppressed (blue) visual neurons in monkeys Da, Eu, Jo, and X. Marker size indicates number of neurons detected at each site. Neurons more rostral than 32 mm were identified as area 8b/9. The asterisk identifies one penetration that was in the medial wall. (B) Location of neurons with auditory responses. (C) Composite coronal section combining AP levels 27 to 33 mm for monkey Da showing all facilitated and suppressed visual (top) and auditory (bottom) responses. The lateral penetration in the premotor eye–ear field encountering only auditory responses is indicated. The medial penetrations passing through pre-SMA are also indicated. All responses that meet visual criterion for baseline deviation were incorporated. The grid is 1 mm.

Neurons with visual responses were concentrated between 27 and 33 mm rostral to the interaural line and between 2 and 5 mm from the midline in SEF (Fig. 12A). In some sites, we found no suppressed responses and only facilitated responses. The lower incidence or absence of visual responses in some penetrations indicated lateral, medial, and rostral boundaries to SEF. In monkey Jo, facilitated visual neurons were identified rostral to SEF, in prefrontal area 8b or 9. As described earlier, these neurons exhibited longer latency and only sustained responses with no pre-excitatory pause. One neuron with a suppressed response like a pause-rebound neuron was found at the border between SEF and area 8b/9. Neurons with auditory responses were also concentrated around 27 to 33 mm rostral to the interaural line and around 2 to 5 mm from the midline in SEF (Fig. 12B). Facilitated neurons were found in all monkeys, while suppressed neurons were found more frequently in monkey Da. No auditory responsive neurons were observed in area 8b/9 of monkey Jo.

Figure 12C shows the depth of all visual and auditory neurons across coronal sections spanning 27 to 33 mm rostral to the interaural line for monkey Da. Visual and auditory neurons were commonly found in SEF on the dorsomedial convexity. A more lateral penetration encountered only auditory neurons. We surmise this is in the premotor eye–ear field lateral to SEF. More medial penetrations found isolated units over a distance >2 mm and fewer visual and auditory neurons. We surmise this is in the skeletal motor pre-SMA (area F6).

#### Laminar depth profile of visual single units

**Figure 13:**
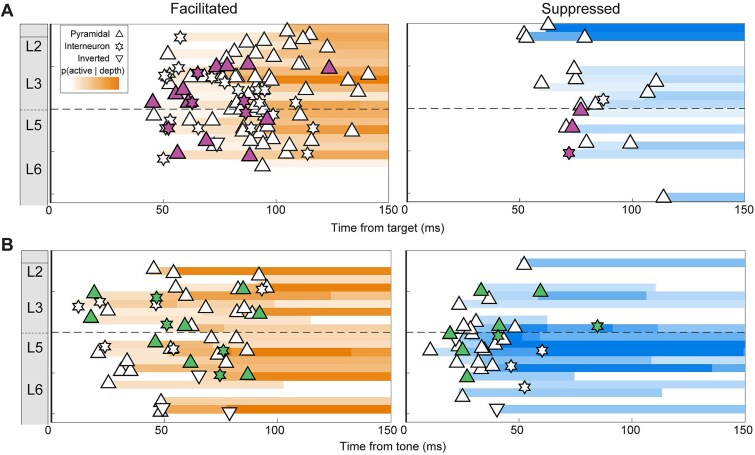
Time–depth plots. (A) Depths of putative pyramidal (triangle), interneuron (star), or inverted (inverted triangle) spikes are plotted as a function of latencies of facilitated (left) or suppressed (right) responses with duration of response indicated by shading. Dashed horizontal line marks boundary between layers 3 and 5. The lower boundary of L6 is not discrete. Unimodal visual responses are white. Bimodal visual and auditory responses are purple. (B) Corresponding plot for auditory responses. Unimodal auditory responses are white. Bimodal visual and auditory responses are green.

Aligning the depths of penetrations across sessions and monkeys enabled reporting average trends. Time–depth profiles of facilitated and suppressed visual and auditory responses were constructed (Fig. 13). In 16 recording sessions for monkeys Eu and X, penetrations were perpendicular to the cortical layers, verified through combined MR and CT imaging (Godlove et al. 2014). Having gained more experience interpreting the laminar organization of single-unit activity in dorsomedial frontal cortex, we included another 12 sessions from monkeys Eu and X in which the penetrations were not perfectly perpendicular. Single units from another 37 sessions were included from monkeys Da and Jo.

Facilitated visual responses were observed in all cortical layers for pyramidal neurons and in L3 and L5/6 for interneurons (Fig. 13A) with hemifield laterality distributed uniformly across layers (Fig. S6). Transient visual responses were observed earlier than sustained responses and concentrated in L3 (Fig. S7). Suppressed visual responses were less common and were nearly all pyramidal neurons in L2/3 and L5. Facilitated visual neurons with auditory responses were both pyramidal and interneurons in L3 and L5/6. The few suppressed visual neurons with auditory responses were only in L5/6. The latencies of facilitated visual responses did not vary across layers or biophysical type, arising ~ 50 ms after target presentation and persisting for at least 150 ms. The latencies of suppressed visual responses tended to be longer in all layers.

#### Laminar depth profile of auditory single units

Facilitated auditory responses were observed in all cortical layers for pyramidal neurons and in L3 and L5/6 for interneurons (Fig. 13B). Suppressed auditory responses were equally common and were predominantly pyramidal neurons in all layers with interneurons restricted to L5/6. Facilitated auditory neurons with visual responses were both pyramidal and interneurons in L3 and L5/6. Suppressed auditory neurons with visual responses were both pyramidal and interneurons concentrated in L3 and L5. As expected, the latencies of auditory responses were shorter than those of visual responses. The latencies of facilitated auditory responses appeared shorter in L3 and L5 than in L2 and L6, arising ~ 20 ms after the tone and persisting for at least 150 ms. The shortest latency responses were observed in both pyramidal and interneurons, but longer latency responses were produced effectively only by pyramidal neurons. The latencies of suppressed auditory responses were also shorter in L3 and L5 than in L2 and L6 but tended to be later than the facilitated responses. Pyramidal neurons tended to have shorter suppressed latencies than did interneurons.

#### Laminar profile of visually elicited current source density

**Figure 14:**
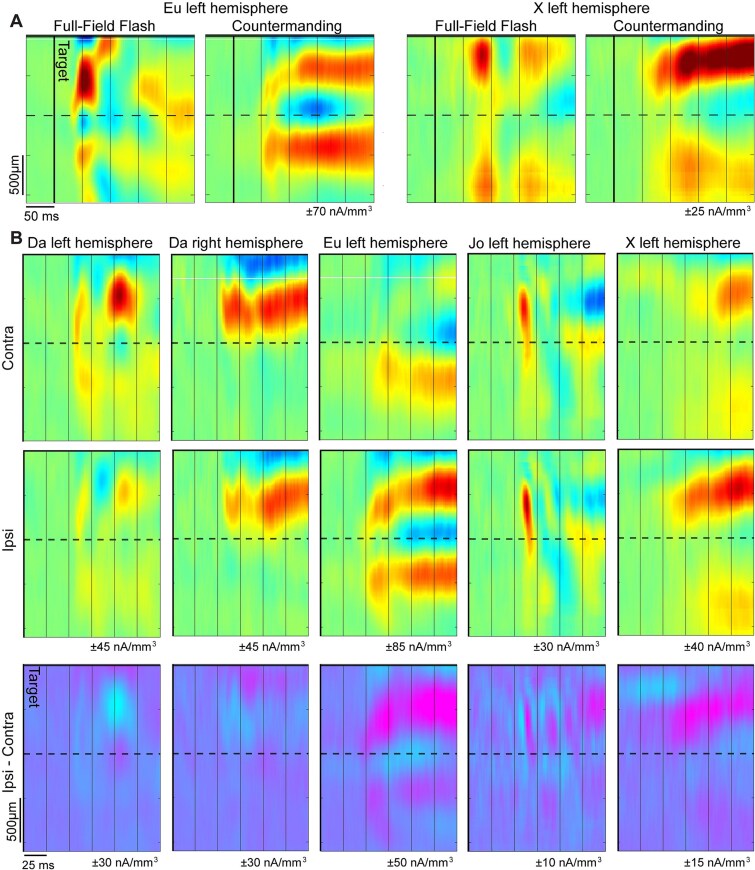
Variation of current-source density with target laterality and stimulus conditions. (A) Grand-averaged visually evoked CSD in response to a full-field flash (left) and during the countermanding task (right) for monkeys Eu and X. Both tasks are shown with the same CSD scale provided at the bottom of countermanding panel. Sinks in the CSD are denoted by red hues, and sources are denoted by blue hues. Dashed horizontal line marks boundary between layers 3 and 5. (B) Grand-averaged visually evoked CSD for contralateral (top), ipsilateral (middle), and their difference (bottom) for each monkey. Monkey Da has recordings from left (L) and right (R) hemispheres. Dashed horizontal line marks boundary between layers 3 and 5. Sinks in the contralateral and ipsilateral CSDs are denoted by red hues, and sources are denoted by blue hues. For the difference in CSDs, negative differences are denoted with a blue hue and positive differences are denoted with a purple hue.

The laminar organization of visual sensitivity was further characterized by calculating the CSD from the average local field potentials in response to visual stimuli (Fig. 14). We report results from the left and from the right hemisphere of monkey Da and from the left hemisphere of Eu, Jo, and X. Several observations merit consideration.

First, structured CSD was evident in each monkey. Being an agranular frontal area, this observation was not guaranteed. However, idiosyncratic variability in the CSD patterns across monkeys and hemispheres was evident. This variability exceeds what has been observed in primary sensory areas. In each monkey, the earliest sink was observed in L3. The initial CSD sink in left hemisphere of Da and in Jo was brief and spanned L3. The initial CSD sink in the right hemisphere of Da and Eu lasted longer but was interrupted briefly before the subsequent prolonged phase. The prolonged current sink in L2/3 in the right hemisphere of Da and in Eu and X became progressively more superficial. For monkey Jo, the early CSD in L3 was not followed by the prolonged sink. The appearance of a brief initial sink throughout L3 in the left hemisphere of Da and in Jo was distinct from the appearance of the other hemisphere and monkeys. In L5/6, the variability in appearance of sinks exceeded that in L2/3. For example, in L5/L6, only monkey Eu exhibited a pronounced, prolonged sink in response to the task target whereas only weak, transient sinks were observed in left hemisphere of Da, in Jo, and in X, while in the right hemisphere of Da no current sink was observed. The basis of this variation requires further investigation. We cannot rule out the hypothesis that these differences represent columnar specialization.

Second, our previous description of visually evoked CSD in SEF was obtained with bright, full-screen flashes (Godlove et al. 2014). Here, we report the laminar structure of CSD in response to the small, task-relevant target. Data from two monkeys (Eu, X) afforded a direct comparison (Fig. 14A) using the depth alignment approach utilized here. The CSDs of individual sessions with passively viewed flashes are provided in Fig. S8. The initial sinks appeared at similar depths in deep L3 and in L5 across stimulus conditions, appearing slightly earlier after the full-screen flash. The most pronounced difference was the prolonged, stronger sinks in upper L3 and L5/6 during the countermanding task relative to the passive flash.

Third, differences were evident between the CSD elicited by contra- and ipsilateral targets (Fig. 14B). Consistent with previous descriptions of a stronger response to stimuli in the contralateral hemifield (eg Schall 1991a), the CSD observed in both hemispheres of monkey Da were stronger for contra- relative to ipsilateral targets. Surprisingly, for monkeys Eu and X the CSD elicited by contralateral targets was weaker than that for ipsilateral targets. This was most pronounced in L3. For monkey Jo, the CSDs elicited by contra- and ipsilateral targets were more similar in strength. The diversity of patterns of CSD observed in penetrations within SEF exceeds what has been reported in sensory areas like V1 (Maier et al. 2010) and V4 (Schroeder et al. 1998; Westerberg et al. 2019). The basis of this diversity requires further investigation.

#### Laminar profile of auditory elicited current-source density

**Figure 15:**
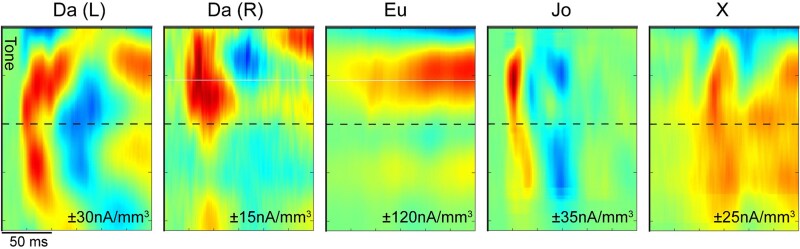
Auditory evoked current-source density for all monkeys. Grand-averaged CSD for each monkey aligned on tone onset. Sinks in the CSD are denoted by red hues, and sources are denoted by blue hues. Dashed horizontal line marks boundary between layers 3 and 5.

We calculated CSD aligned on the tone for each monkey (Fig. 15 and Fig.  S9). While resembling visually evoked CSDs, several differences were observed. First, the initial sinks were earlier at ~ 25 ms in all monkeys and between hemispheres in monkey Da. Second, notable differences were evident across monkeys and hemispheres. The left hemisphere of Da and Jo exhibited transient initial sinks which appeared to span L3, L5, and most or all of L6. This extended sink was not observed in the right hemisphere of Da which exhibited a single large sink restricted to L2/3 and another sink in L6. Eu and X exhibited more prolonged sinks. Like that in the right hemisphere of Da, the CSD in Eu was absent in L5/6.

## Discussion

These results provide the first description of the laminar organization of task-related visual and auditory responsiveness in SEF, complementing our previous description of the laminar organization of responses to passive visual flashes (Godlove et al. 2014), neurons’ signaling errors and reward gains and losses (Sajad et al. 2019), and neurons’ signaling conflict, event timing, and goal maintenance (Sajad et al. 2022). These findings have reinforced the basic differences between granular sensory and agranular cortical areas (Ninomiya et al. 2015).

### Visual responses in the supplementary eye field

Around 24% of neurons recorded from SEF responded to presentation of visual targets. Previous studies have reported between ~ 10% and 70% of visually responsive neurons (Schlag and Schlag-Rey 1987; Schall 1991a, 1991b; Russo and Bruce 2000; Roesch and Olson 2003; Nakamura et al. 2005; Pouget et al. 2005; Purcell et al. 2012; Godlove et al. 2014; Bharmauria et al. 2021). Previous studies have also demonstrated both facilitation and suppression (Schlag and Schlag-Rey 1987; Schall 1991a). However, consistent with Schall (1991a), we observed relatively few visually suppressed neurons. This contrasts with Godlove et al. (2014), who reported that 50% of neurons were suppressed in response to bright, full-screen flash. Differences across studies are likely due to differences in single-unit sampling procedures, visual stimulus properties, strategies for characterizing neurons based on research goals, and statistical inclusion criterion. Another source of differences across studies can be the depth of sampling. Our linear electrode array samples across all layers found visually responsive neurons across all layers of SEF.

The visual responses in SEF can be delivered by afferents from the frontal lobe (frontal eye field and adjacent prefrontal areas), the parietal lobe (lateral intraparietal area and area 7a), and the temporal lobe (medial superior temporal area and superior temporal polysensory area) (Maunsell and van Essen 1983; Andersen et al. 1990; Huerta and Kaas 1990; Schall et al. 1993). Another possible source of afferents is from the lateral sector of the mediodorsal nucleus of the thalamus (Huerta and Kaas 1990; Shook et al. 1991), which conveys visual signals to the frontal eye field (Sommer and Wurtz 2004). However, the nature of the signals conveyed from the mediodorsal nucleus to SEF are unknown. SEF is innervated by other thalamic nuclei, including the ventral anterior nucleus, nucleus X, the posterior subdivision of the ventral lateral nucleus, the central lateral nucleus, the parafascicular nucleus, and the suprageniculate-limitans nucleus (Matelli et al. 1989; Huerta and Kaas 1990; Shook et al. 1991), but the visual responsiveness of neurons in those nuclei is untested.

The visual response latencies were generally consistent with those reported previously (Pouget et al. 2005; Godlove et al. 2014). The Godlove study measured a higher fraction of shorter latency responses, but these were in response to the brightest flash of the entire computer monitor. The values from the Pouget study were obtained under stimulus conditions corresponding to the present study. The visual response latencies in SEF follow those in V1, MT/MST, and FEF (Schmolesky et al. 1998). The long latency and sustained responses of most SEF neurons are consistent with input from slower pathways. However, the fast, transient responses of some SEF neurons are likely to arise from faster pathways through MT, MST, and FEF. Our finding that neurons with short-latency, transient responses tended to be located in L2/3 implies that afferents from areas MT and MST terminate in the upper layers of SEF.

The fast transient responses can contribute to the transient suppression measured as the pre-excitatory pause. However, the pre-excitatory pause of some neurons was measured before target presentation, which entails an explanation involving processes predicting target presentation. To minimize temporal expectation, the interval between fixation of the central spot and presentation of the target was sampled from a non-aging distribution with a minimum value of 1,000 ms and truncated at 1,700 ms such that the hazard rate of target presentation was constant until the last ~ 50 ms of the interval. Nevertheless, progressive increases or decreases in discharge rates can be seen in the representative neurons illustrated in Figs. 5A, 6, and  9, as described in previous studies (eg Schall 1991b; Coe et al. 2002). The characteristics of predictive neural modulation in SEF during this task merit further research.

We also identified neurons described as pause-rebound. They are suppressed by the visual target and are reactivated with saccade initiation. This profile aligns with fixation cells described in the superior colliculus and frontal eye field by other researchers. However, because we did not perform tests necessary to distinguish sensory from motor factors, we do not conclude that these are indeed fixation neurons.

Facilitated responses of putative interneurons tended to be earlier than those of putative pyramidal neurons. This observation, which is consistent with other observations in the frontal lobe (Diester and Nieder 2008), allows for a possible role in feedforward inhibition (Swadlow 2003), which can be generated by larger and faster excitatory–postsynaptic potentials in interneurons compared to pyramidal neurons as well as faster channel kinetics.

The present sample of visual response latencies in SEF were indistinguishable from those measured previously in cingulate cortex (Pouget et al. 2005). However, that publication reported marginally but significantly shorter latencies in SEF compared to cingulate cortex. Variation in latencies sampled across monkeys may explain the difference of outcomes, so resolution of this comparison between dorsomedial and cingulate cortex visual latencies will require larger sampling of both regions simultaneously under matched conditions.

Previous studies of SEF visual responses have found that receptive fields are most common in the contralateral hemifield (Schall 1991a, 1991b; Russo and Bruce 2000; Purcell et al. 2012), but the ipsilateral representation exceeds that in FEF (Schall 1991a, 1991b). Consequently, we were surprised to find the preponderance of neurons with stronger responses to ipsilateral targets. They could be observed here because we sampled spikes across all cortical layers with linear multi-electrode arrays after sufficient time to allow stabilization of the tissue and abeyance of spreading depression. Ipsilateral visual hemifield preference might also have been overlooked due to differences in visual stimulus arrangement and task demands. For instance, Purcell et al. (2012) noted that the mean response of SEF neurons was reduced to a visual search array relative to a single stimulus (see also Nakamura et al. (2005)). This may have resulted from suppression across the visual field. Also, Roesch and Olson (2003) observed that changing the reward amount altered visual responsiveness as well as directionality preference. The presence of stronger ipsilateral responses in SEF can be explained by the pronounced connections of SEF with the SEF and FEF in the other hemisphere (Huerta et al. 1987; Huerta and Kaas 1990) plus inputs from neurons representing both hemifields in parietal area 7a (Motter and Mountcastle 1981) and the superior temporal polysensory area (Bruce et al. 1981). Additionally, the prefrontal cortex, which has extensive reciprocal connections with SEF (Huerta and Kaas 1990), also responds to visual information in the ipsilateral hemifield (Wimmer et al. 2016).

We observed differences between laminar CSD profiles between monkeys. Such differences can be due to sex and species differences. Heterogeneity across SEF is another possible explanation, which is consistent with differences in the neuron types found in different cortical columns (Sajad et al. 2019; Sajad et al. 2022). Another salient observation was the prolongation of the initial upper and lower sinks present after the onset of visual stimuli in the countermanding task compared to the full-field flash. While it is apparent that in both tasks initial input is received in similar layers, the CSD was notably prolonged, merging the individual sinks found in the full-field flash task together. This transition may highlight a shift in SEF from feedforward to local recurrent processing (Douglas and Martin 2004). Additionally, this persistent activity may be driven by slow kinetics of NMDA which maintain persistent activity in prefrontal cortex during working memory (Compte et al. 2000; Wang 2001).

### Auditory responses in the supplementary eye field

Around 26% of neurons recorded from SEF responded to presentation of auditory tones. As an area heavily involved in the higher-order visual pathway and voluntary saccade generation, its role in processing auditory responses has not been well explored. The anatomical pathways delivering auditory signals to SEF are less well characterized. Potential sources include afferents from the superior temporal polysensory area (Bruce et al. 1981) and polymodal regions of parietal and prefrontal cortex (Huerta and Kaas 1990) that are innervated by auditory cortex (Camalier et al. 2012). Another possible source is a region lateral to SEF in area 8B, referred to as a premotor ear–eye field (Lanzilotto et al. 2013), which projects robustly to SEF (Luppino et al. 2003; Wang et al. 2005). In fact, an electrode penetration in this region identified only auditory and no visual neurons.

The auditory latencies we measured were later than those in auditory cortex (Camalier et al. 2012) but were earlier than the visual response latencies, consistent with the faster processing observed in the auditory compared to the visual system. The CSD evoked by the tone differed from the pattern observed in primary auditory cortex of macaques where the initial sinks appear in L4 at a latency of ~ 10 ms (Steinschneider et al. 1992; Kajikawa and Schroeder 2011; O’Connell et al. 2014). Some investigators describe additional sinks in lower L3 and in L5 (Kajikawa and Schroeder 2011; O’Connell et al. 2014).

## Conclusion

This description of the laminar structure of visual and auditory responses during a countermanding saccade task offers new information about the functional architecture of SEF. The findings complement and contrast with an earlier description of the passive visual responses in SEF (Godlove et al. 2014) and highlight further differences between agranular frontal cortex and the canonical cortical microcircuit derived from primary visual cortex (Ninomiya et al. 2015). The findings complement our previous reports of the laminar organization of neurons involved with performance and reward monitoring (Sajad et al. 2019) as well as conflict, event timing, and goal maintenance (Sajad et al. 2022) and complete the first draft of the functional architecture of an agranular frontal area.

## Supplementary Material

Thirunavukkarasu_Errington_Sajad_Schall-Supplemental_Figures-ACCEPTED_bhag064
