# Supplementary material for: Laminar architecture of visual and auditory responses in the supplementary eye field of macaques
Source: Cereb Cortex. 2026 Jun 19;36(6):bhag064. doi: 10.1093/cercor/bhag064 (PMC13280951; doi:10.1093/cercor/bhag064)
Supplement: Thirunavukkarasu_Errington_Sajad_Schall-Supplemental_Figures-ACCEPTED_bhag064 [file thirunavukkarasu_errington_sajad_schall-supplemental_figures-accepted_bhag064.pdf]

Supplementary Figures

Laminar architecture of visual and auditory responses in supplementary eye field of macaques

Pranavan Thirunavukkarasu[1], Steven P. Errington[2], Amirsaman Sajad[3], Benjamin W. Corrigan[1], Jeffrey D. Schall[1,*]

[1] Centre for Integrative and Applied Neuroscience, Centre for Vision Research, Department of Biology, York University, Toronto, ON M3J 1P3 , Canada

[2] Biosciences Institute, Faculty of Medical Sciences, Newcastle University, Newcastle upon Tyne NE1 7RU, United Kingdom

[3] International Center for Primate Brain Research, Center for Excellence in Brain Science and Intelligence Technology, Chinese Academy of Sciences, Shanghai 201602, P.R. China

***Lead Contact and Corresponding Author:**

Jeffrey D. Schall, Ph.D.
Department of Biology, York University, Toronto, ON M3J 1P3 , Canada
schalljd@yorku.ca

Running title: Functional architecture of sensory responses in frontal lobe

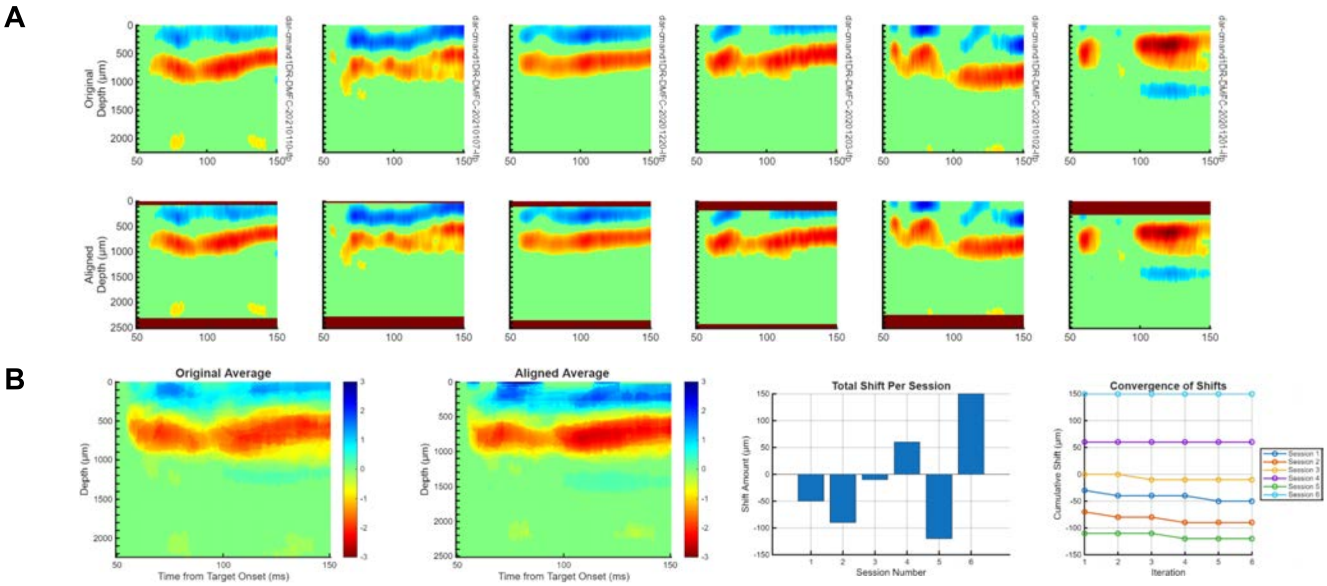

Figure S1. Automated Depth Alignment Procedure. **A.** Figure shows alignment procedure used to align depths across sessions from the right hemisphere of monkey Da in Fig. 4. Top row contains CSDs for sessions spanning across a rough estimate of grey matter bounds obtained through physiological measures (Fig. 3). Bottom row contains results of fine-tuned alignment procedure. **B.** Two panels on left show the average aligned session before and after alignment. The two right panels show the total shifts applied at the end of convergence and the progression of shifts across iterations for all the sessions respectively.

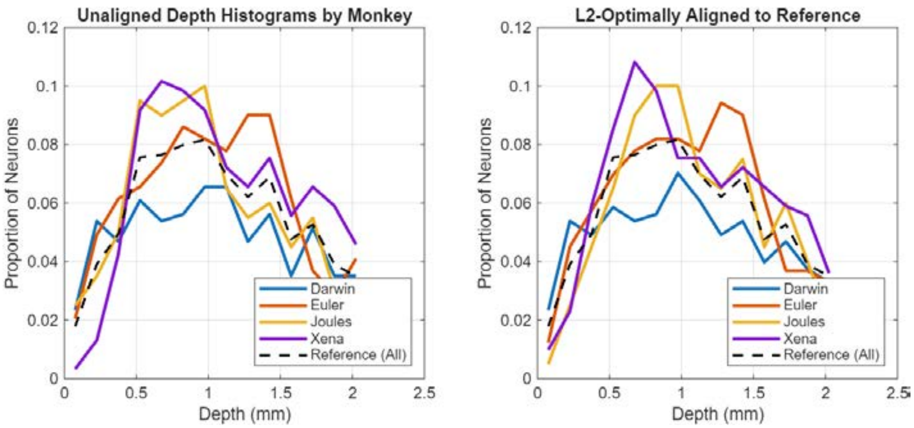

Figure S2. Aligning SEF Sessions Between Animals Using Laminar Proportion of Neurons. Left panel contains neuronal laminar proportion from CSD-aligned sessions between animals. Right panel alignment was achieved by applying small depth shifts that minimized the squared error relative to a pooled reference distribution of neuronal depths.

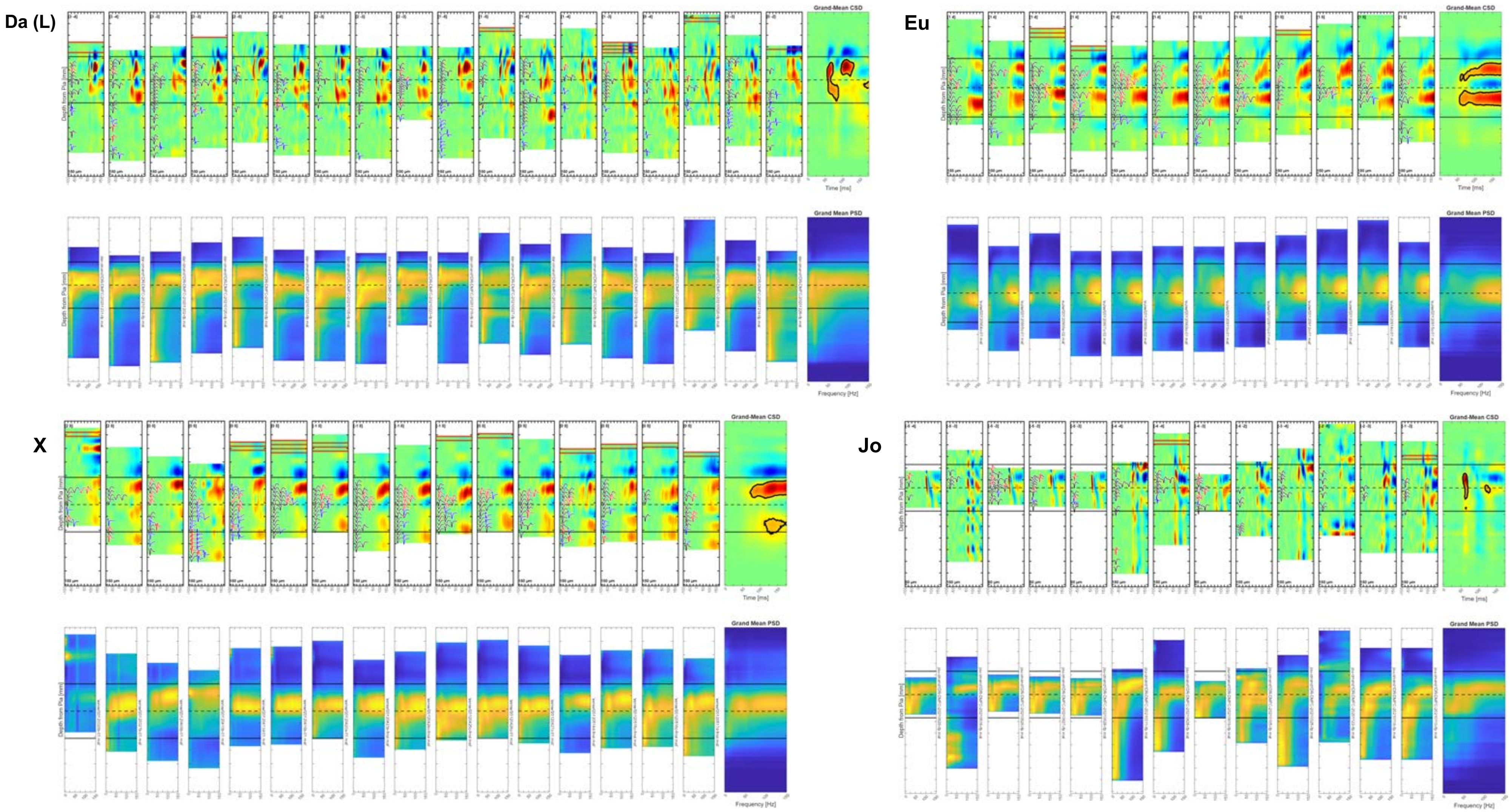

Figure S3. Results of session alignment across SEF in monkey Da (Left Hemisphere), Eu, Jo and X. Results from Da (Right Hemisphere) are shown in **Fig. 4.** CSDs for individual sessions shown here are Z-score normalized ranging between [-5 5] and the CSD for the grand-averaged CSDs shown here are calculated from the average of Z-scored CSDs ranging from [-4 4].

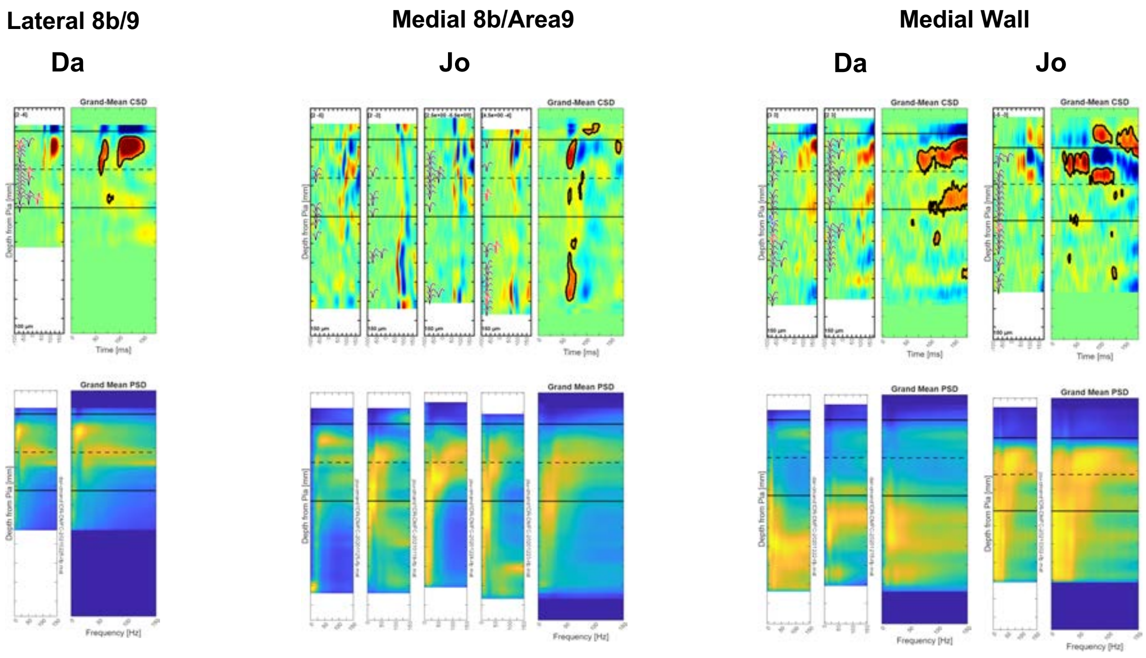

Figure S4. Results of session alignment across area bordering laterally (lateral Area 8b), rostrally (Medial Area 8b/9) and medially to SEF (medial wall). CSDs shown are with respect to array onset. CSDs for individual sessions shown here are Z-score normalized ranging between [-5 5] and the CSD for the grand-averaged CSDs shown here are calculated from the average of Z-scored CSDs ranging from [-4 4].

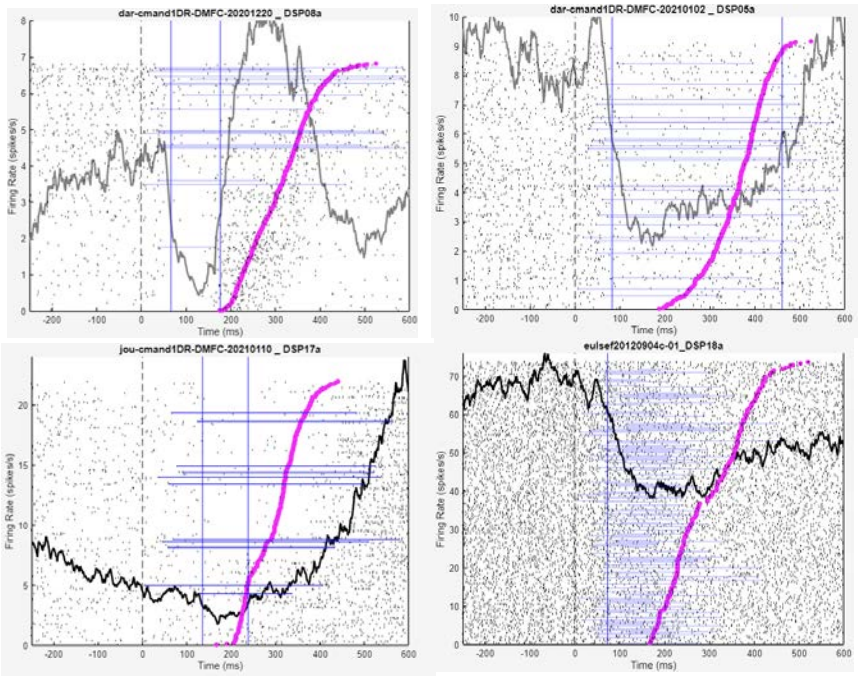

Figure S5. Additional examples of pause-rebound neurons with more sluggish response to target presentation and saccade production. Each row of rasters signifies a different trial with magenta dots indicating saccade latencies and blue bars indicating significant burst suppression periods.

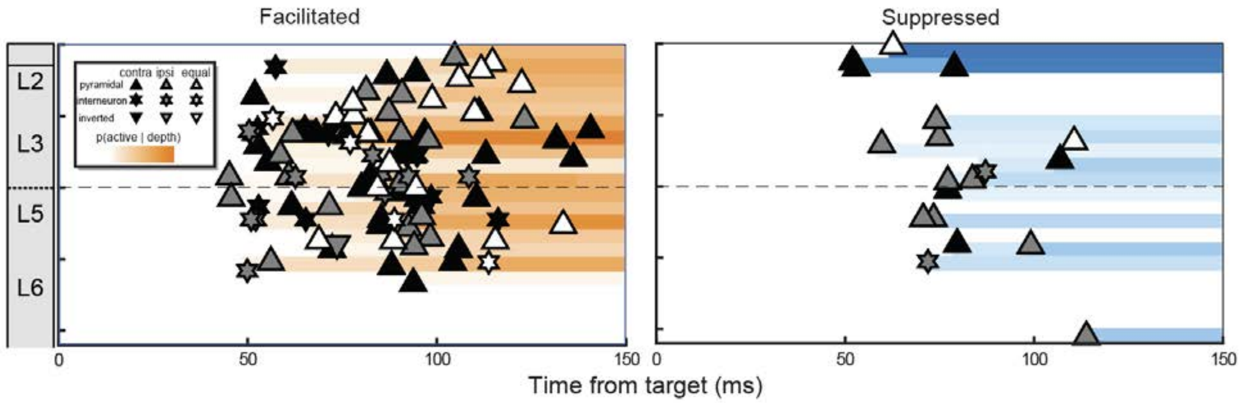

Figure S6. Time-depth plots of visual responses by laterality preference. A. Time-depth plot showing latency and recruitment of facilitated (left) and suppressed (right) visual responses across depth for neurons recorded from penetrations perpendicular to the layers. Symbols mark beginning of visual response for neurons with broad (triangles) and narrow (stars) spike widths with stronger contralateral (black), ipsilateral (gray), or balanced (white) responses. Color maps indicate the percentage of neurons with visual responsiveness through time at each depth relative to the sampling distribution of all well-isolated neurons. Dashed horizontal line marks boundary between layers 3 and 5. The lower boundary of L6 is not discrete.

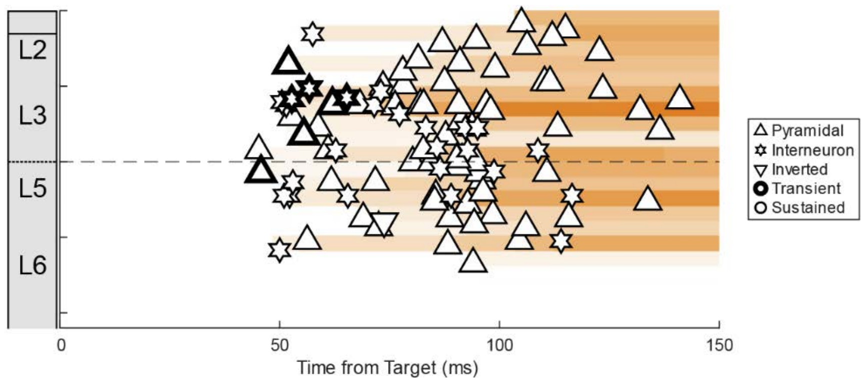

Figure S7. Time-depth plots of facilitated transient and sustained visual responses. A. Time-depth plot showing latency and recruitment of facilitated visual responses across depth for neurons recorded from penetrations perpendicular to the layers. Symbols mark beginning of visual response for neurons with broad (triangles) and narrow (stars) spike widths with transient responses bolded in black. Color maps indicate the percentage of neurons with visual responsiveness through time at each depth relative to the sampling distribution of all well-isolated neurons. Dashed horizontal line marks boundary between layers 3 and 5. The lower boundary of L6 is not discrete.

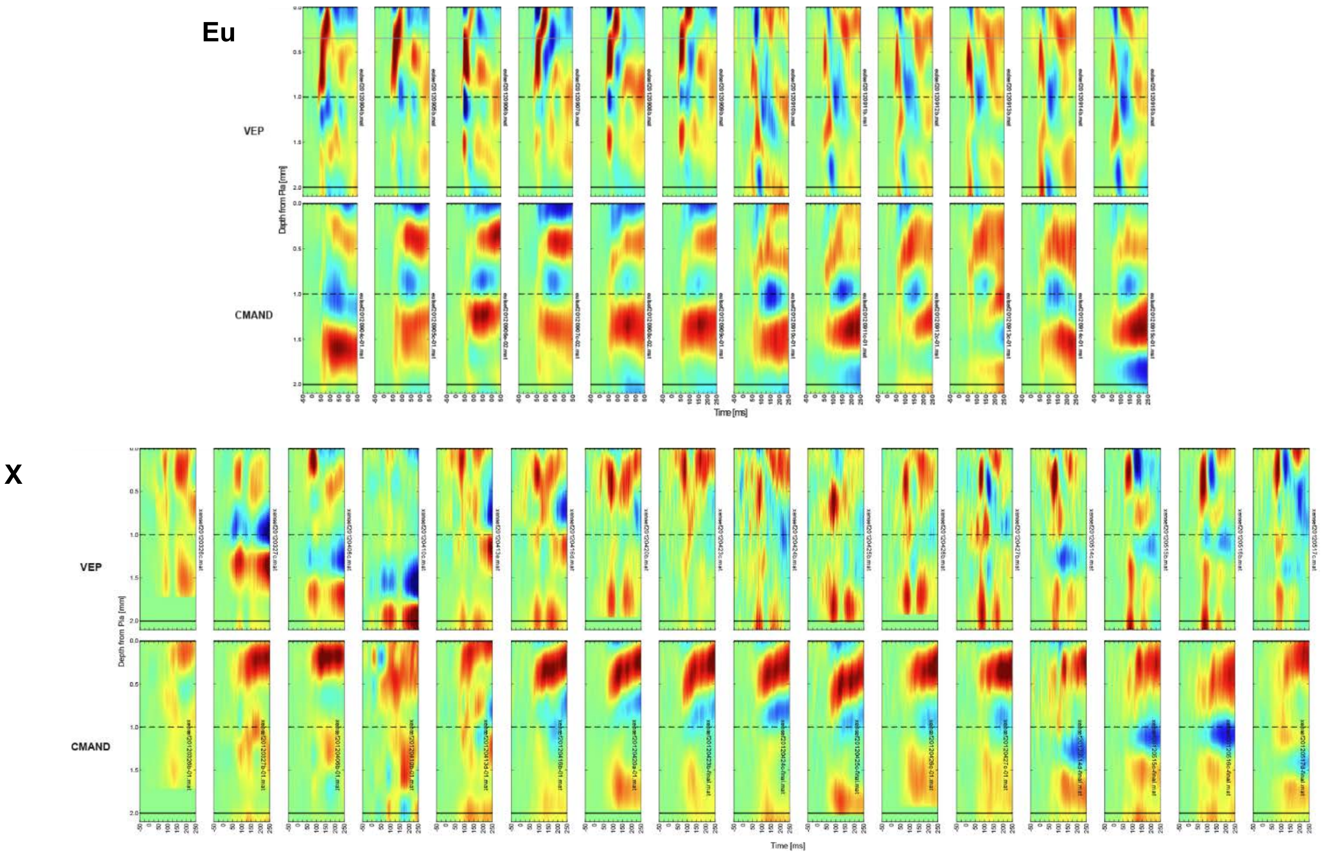

Figure S8. Time-depth plots of sensory responses. Individual sessions of visually- evoked (VEP) CSD in response to a full field flash (top) and during the countermanding task (bottom). CSDs are standardized between ±4SD.

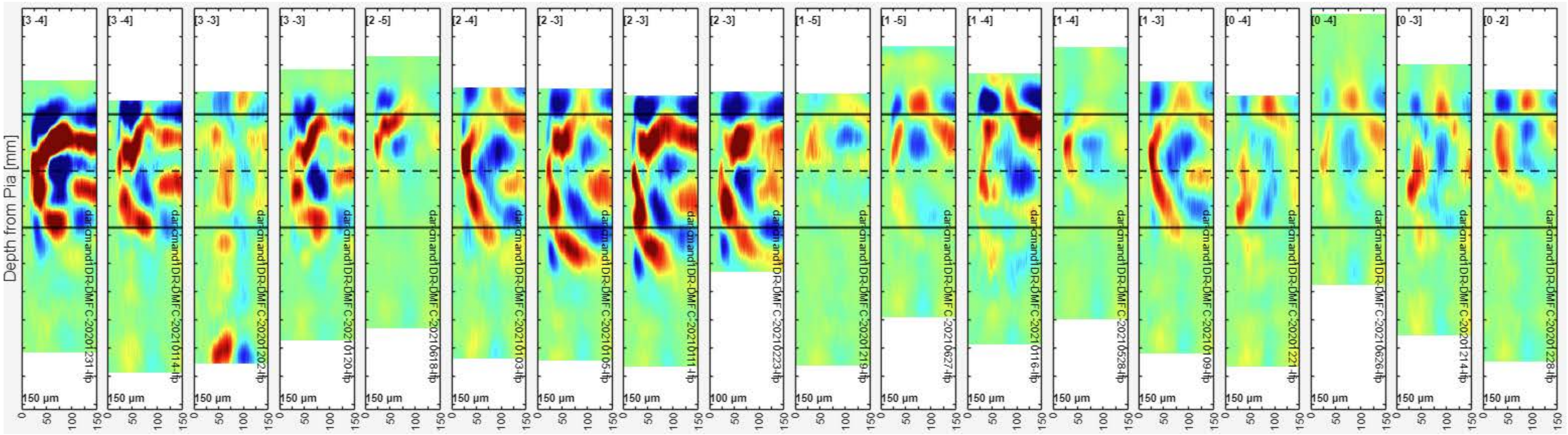

Figure S9. Tone-aligned session recorded from SEF in monkey Da (Left Hemisphere). CSDs for individual sessions shown here are Z-score normalized ranging between [-5 5]
